# Relative role of border restrictions, case finding and contact tracing in controlling SARS-CoV-2 in the presence of undetected transmission: a mathematical modelling study

**DOI:** 10.1186/s12916-023-02802-0

**Published:** 2023-03-16

**Authors:** Rachael Pung, Hannah E. Clapham, Timothy W. Russell, CMMID COVID-19 Working Group, Vernon J. Lee, Adam J. Kucharski

**Affiliations:** 1 Ministry of Health, Singapore, Singapore; 2 Centre for the Mathematical Modelling of Infectious Diseases, London School of Hygiene and Tropical Medicine, London, UK; 3 Saw Swee Hock School of Public Health, National University of Singapore, Singapore, Singapore

**Keywords:** Border restrictions, Case finding, Contact tracing, Mathematical modelling, SARS-CoV-2, Undetected

## Abstract

**Background:**

Understanding the overall effectiveness of non-pharmaceutical interventions to control the COVID-19 pandemic and reduce the burden of disease is crucial for future pandemic planning. However, quantifying the effectiveness of specific control measures and the extent of missed infections, in the absence of early large-scale serological surveys or random community testing, has remained challenging.

**Methods:**

Combining data on notified local COVID-19 cases with known and unknown sources of infections in Singapore with a branching process model, we reconstructed the incidence of missed infections during the early phase of the wild-type SARS-CoV-2 and Delta variant transmission. We then estimated the relative effectiveness of border control measures, case finding and contact tracing when there was no or low vaccine coverage in the population. We compared the risk of ICU admission and death between the wild-type SARS-CoV-2 and the Delta variant in notified cases and all infections.

**Results:**

We estimated strict border control measures were associated with 0.2 (95% credible intervals, CrI 0.04–0.8) missed imported infections per notified case between July and December 2020, a decline from around 1 missed imported infection per notified case in the early phases of the pandemic. Contact tracing was estimated to identify 78% (95% CrI 62–93%) of the secondary infections generated by notified cases before the partial lockdown in Apr 2020, but this declined to 63% (95% CrI 56–71%) during the lockdown and rebounded to 78% (95% CrI 58–94%) during reopening in Jul 2020. The contribution of contact tracing towards overall outbreak control also hinges on ability to find cases with unknown sources of infection: 42% (95% CrI 12–84%) of such cases were found prior to the lockdown; 10% (95% CrI 7–15%) during the lockdown; 47% (95% CrI 17–85%) during reopening, due to increased testing capacity and health-seeking behaviour. We estimated around 63% (95% CrI 49–78%) of the wild-type SARS-CoV-2 infections were undetected during 2020 and around 70% (95% CrI 49–91%) for the Delta variant in 2021.

**Conclusions:**

Combining models with case linkage data enables evaluation of the effectiveness of different components of outbreak control measures, and provides more reliable situational awareness when some cases are missed. Using such approaches for early identification of the weakest link in containment efforts could help policy makers to better redirect limited resources to strengthen outbreak control.

**Supplementary Information:**

The online version contains supplementary material available at 10.1186/s12916-023-02802-0.

## Background

The use of multiple outbreak control measures in the early phases of the COVID-19 pandemic was resource intensive and disruptive, but essential to minimise the loss of lives [1, 2]. Measures such as case finding at the borders and healthcare touchpoints allow health authorities to assess the extent of disease importation and undetected spread in the community. Furthermore, contact tracing around notified cases can identify potential transmission routes and hence new cases [3, 4]. When multiple control measures are implemented together, understanding the effectiveness of each measure enables public health authorities to focus on the most effective measures when resources are limited and to minimise interruption to economic and social activities. Studies typically evaluate the collective effectiveness of country or region-specific COVID-19 outbreak control measures by measuring changes to the reproduction number using overall observed case incidence [1, 5–12] or only focus on the impact of specific interventions using outbreak data [13, 14]. If analysis could disentangle the observed and unobserved transmission dynamics, it would therefore be possible to obtain higher resolution insights on the effects of each outbreak control measure.

Transmission chains from outbreak clusters have been used to characterise the reproduction number of infectious diseases other than COVID-19 and the relative contribution of different transmission routes (e.g. imported or environmental introduction vs community) to the overall spread [15–18]. However, these studies typically do not account for the role of missed infections (e.g. asymptomatic or mildly symptomatic infections) in influencing the effectiveness of outbreak control measures. To our knowledge, the use of data on these transmission linkages to estimate the burden of infection for SARS-CoV-2 at the population level has yet to be documented. The extent of missed infections in the COVID-19 pandemic was commonly assessed via population-wide seroprevalence surveys [19, 20], excess mortality studies [21], random community testing [22] or behavioural surveys [23, 24]. However, during the initial phases of an outbreak of a novel pathogen, serological assays to measure the disease prevalence are generally not available. Moreover, these methods do not provide assessment on the extent of missed cases at the borders. Thus, methods to address these challenges and provide a more complete view of the outbreak are necessary.

With a population of 5.7 million inhabitants, Singapore was one of the first countries to report SARS-CoV-2 infections outside of mainland China at the beginning of the COVID-19 pandemic. The Ministry of Health monitored the daily incidence of imported, and linked and unlinked local COVID-19 cases and collected extensive information on the epidemiological events associated with each case (e.g. time of arrival, symptoms onset, notification, isolation or quarantine). In this study, we reconstructed the pandemic trajectory in Singapore and estimated the effectiveness of various outbreak control measures (Table 1) by combining the observed COVID-19 cases with a mathematical model. As countries redesign surveillance systems for future pandemics, this modelling framework has the potential to inform how the collection of different data fields can shape our understanding of disease transmission in the early phases of a pandemic.

**Table 1:** Outbreak control measures in Singapore. Observed case data were used to estimate the effectiveness of each measure. Cases are defined as infected individuals that tested positive and are notified, while infections include all notified and missed infected individuals

Control measure *(Aims)*	Description	Observed data (●) and modelled outputs (◆)
Border control *(Minimise disease introduction into community)*	■ Limiting the number of incoming travellers from countries with ongoing outbreaks ■ Quarantine or restricting movement of incoming travellers	◆ Number of missed imported infections
Case finding *(Targeted testing at known or potential source(s) of infection)*	■ Testing of symptomatic travellers upon arrival or when they developed symptoms during quarantine ■ Testing regime for non-symptomatic travellers	● Imported case data
	■ Testing of suspect cases (e.g. persons with clinical signs and symptoms suggestive of pneumonia or severe respiratory infection, persons with acute respiratory infection and travel history to regions with ongoing outbreak) ■ Routine testing of high-risk populations (e.g. healthcare workers, nursing home residents) ■ Ad-hoc testing during cluster outbreak investigations	● Local unlinked case data ◆ Effectiveness of case finding
Contact tracing *(Targeted testing at potential routes of infection)*	■ Interviewing COVID-19 cases or use of Bluetooth contact tracing devices to identify close contacts ■ Testing of symptomatic contacts ■ Testing of contacts before the end of their quarantine	● Local linked case data ◆ Effectiveness of contact tracing
Use of other non-pharmaceutical interventions and vaccines *(Untargeted community- or population-level preventive measures)*	■ Physical distancing ■ School and venue closure ■ Large-scale population movement restrictions and the corresponding need to work-from-home ■ Population-wide face mask usage ■ Pre-event testing/vaccination ■ Accelerated development and roll-out of COVID-19 vaccines (primary doses and boosters) with priority given to frontline workers and the elderly before progressively offered to younger age groups	◆ Average number of secondary cases generated by a single infectious individual over the course of the entire infectious period (i.e. R)

## Methods

### Data

Cases of COVID-19 (confirmed with a respiratory sample positive for SARS-CoV-2 by PCR [25] were identified through case finding and contact tracing (Table 1). Extensive epidemiological investigations were conducted for each case to establish their exposure history and to classify them as a local linked case if a case has at least one known source of infection or a local unlinked case if a case has an unknown source of infection.

In this study, we used COVID-19 cases notified to the Ministry of Health, Singapore from Jan 23 to Dec 31, 2020, and from Apr 1 to Aug 18, 2021, in Singapore. The former time period precedes the detection and surge in cases infected by SARS-CoV-2 Variants of Concern in Singapore [26], while community spread in the latter time period was dominated by the SARS-CoV-2 Delta variant [27]. Data from Jan to Mar 2021 was not used as the COVID-19 incidence in the community was too low (i.e. less than 5 cases per day) for any meaningful analysis.

For the two time periods of study, all confirmed cases were conveyed to secured isolation facilities and discharged after 21 days from the date of confirmation if assessed to be clinically well, or with sequential negative tests. Cases occurring in persons residing in a foreign-worker dormitory and notified from Apr 7 to Oct 31, 2020, were omitted from the analysis as these dormitories were placed under lockdown for an extended period of time. As workers were subjected to movement restrictions, there was a minimal opportunity to interact with the community and hence they were assumed to be incapable of driving community-level transmission. Furthermore, around 0.2% of the confirmed cases occurred in healthcare workers providing care to confirmed cases. As these cases were not community-acquired infections, they were omitted from the analysis.

### Transmission model

**Fig. 1:**
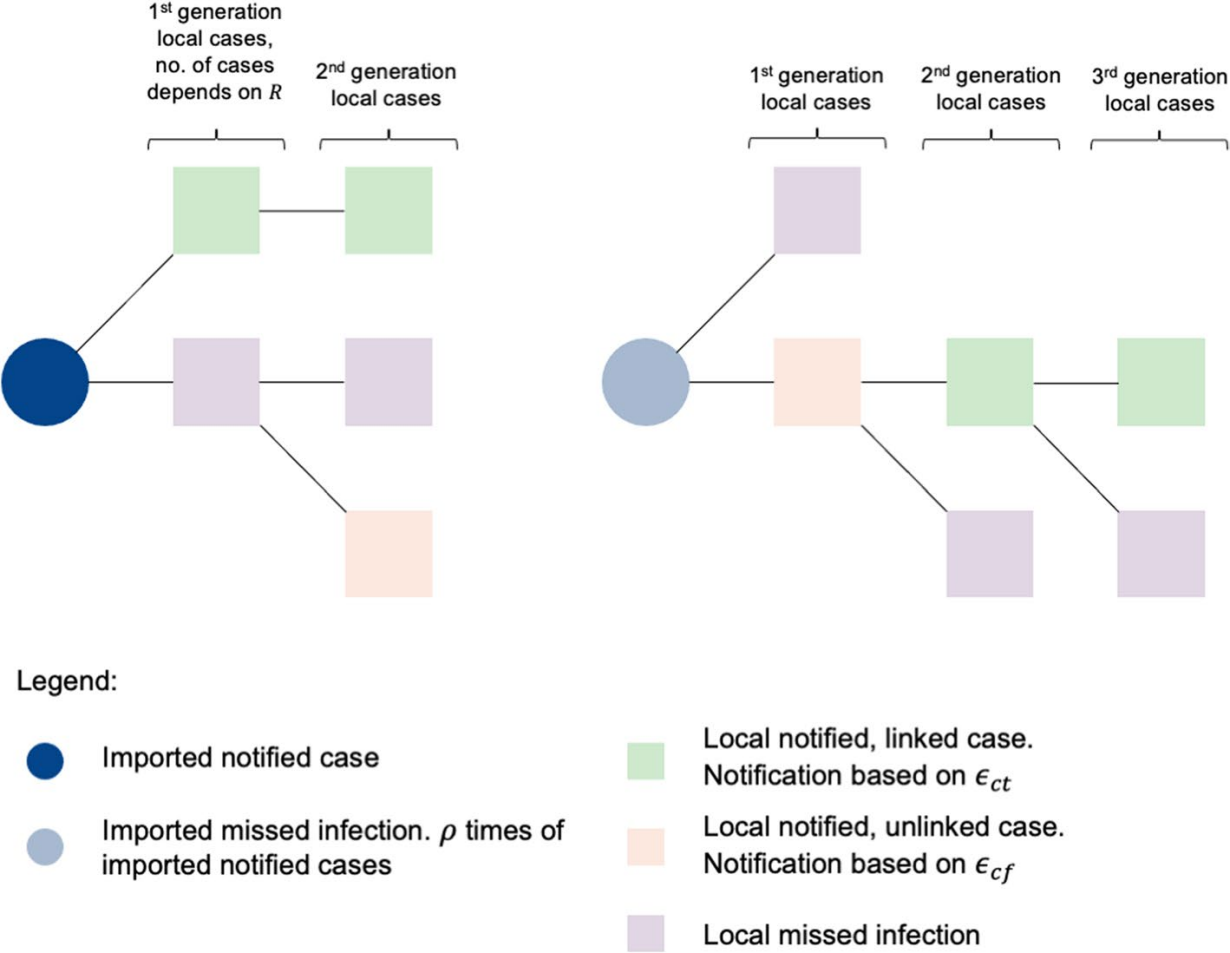
Branching process model and model parameters

Using the notified linked and unlinked cases, we fitted a branching process model using a Bayesian framework to estimate the effectiveness of different control measures (Fig. 1, Table 1 and Additional file 1: Table S1), such as (i) border control measures (based on the extent of missed imported infections, *ρ*), (ii) case finding (*ϵ*_*cf*_), (iii) contact tracing (*ϵ*_*ct*_), (iv) other outbreak control measures (based on the community reproduction number, *R*), and estimate the incidence of missed COVID-19 infections.

For a single infectious individual, the mean rate at which an individual infects others (i.e. infectiousness) *τ* time since infection, *β*(*τ*), can be expressed as a function of the generation interval, *ω*(*τ*) and the reproduction number, *R*:

1 $$\beta \left(\tau \right)=\omega \left(\tau \right)R$$

*ω*(*τ*) is the probability density function of the time from infection in one case to another and is often approximated using serial intervals (i.e. time from symptom onset in one case to another). We modelled *ω*(*τ*) as a lognormal distribution with mean 5.9 and standard deviation 2.4, approximated using published estimates of the observed serial interval for COVID-19 during the early stages of the outbreak when the generation interval and the observed serial interval had yet to reduce substantially due to the influence of non-pharmaceutical interventions [28–30]. With the exception of *ω*(*τ*), all other probability density functions are denoted as *f* in the subsequent sections.

*R* is defined as the average number of secondary cases generated by a single infectious individual over the course of the entire infectious period (i.e. no truncation of the infectious period due to individually-targeted measures such as quarantine or isolation). Furthermore, the effects of various outbreak control measures not related to case finding or contact tracing (e.g. social distancing, vaccination) were collectively modelled within *R* (Table 1).

COVID-19 infections at calendar time *t* were either notified, *n*, or missed, *m*. These infections can be further stratified based on their sources of infection and denoted by subscript *im* for imported infections, *cf* for local unlinked infections identified through case finding, and *ct* for local linked infections identified through contact tracing. Early in the pandemic, COVID-19 was introduced in most countries by the arrival of infectious travellers at time *t* − *a* who could be notified to the public health authorities, *n*_*im*_(*t* − *a*) or missed, *m*_*im*_(*t* − *a*). Beside the time of arrival, the time of symptoms onset, *t* − *s*, of a notified case is often observed but not the time of infection, *t* − *τ*. Estimating the time of infection of notified imported cases would allow us to estimate the potential number of local infections generated by these cases since their time of arrival. Thus, the time series of notified imported cases by the time of infection and arrival is defined as:

2 $${n}_{im}\left(t-\tau, t-a\right)={n}_{im}\left(t-a\right){f}_a\left[\left(t-\tau \right)-\left(t-a\right)\right],\hspace{10mm}for\hspace{2mm}t-\tau \le t-a$$

3 $${f}_a(x)={\int}_0^{\infty }{f}_s(u){f}_{sa}\left(x-u\right)\ du$$

where *f*_*a*_(*x*) is the probability density function of arriving to a country *x* time since infection and *u* is the variable of integration. *f*_*a*_(*x*) is derived, by convolving the incubation period for SARS-CoV-2 infection *x* time since infection, *f*_*s*_(*x*), and the observed distribution of time from symptoms onset to arrival, *f*_*sa*_[(*t* − *a*) − (*t* − *s*)] (Eq. 3). *s* − *a* is the time delay to developing symptoms since arrival and *s* − *a* > 0 implies that case was symptomatic before arrival and vice versa. We modelled *f*_*s*_(*τ*) as a lognormal distribution with mean 5.8 days and standard deviation 3.1 days for wild-type SARS-CoV-2 [31] and mean 4 days and standard deviation 0.4 days for the Delta variant [32].

Missed imported infections were modelled to scale by a factor, *ρ*, of notified imported cases (Eq. 4). Both notified and missed imported infections were capable of generating community infections from their time of arrival to isolation or the end of their infectiousness respectively. Community infections, denoted by subscript *c*, infected on day *t* were either notified, *n*_*c*_(*t*), through varying effectiveness of case finding and contact tracing or missed, *m*_*c*_(*t*) in Eqs. (5) and (6).

4 $${m}_{im}\left(t-\tau, t-a\right)=\rho\ {n}_{im}\left(t-\tau, t-a\right)$$

5 $$\begin{aligned}n_c(t)&=\epsilon_{ct}\int_0^\infty\int_0^\tau n_{im}\left(t-\tau,t-a\right)F_{h'}\left(\tau\right)\beta\left(\tau\right)\;dad\tau+\epsilon_{cf}\int_0^\infty\int_0^\tau m_{im}\left(t-\tau,t-a\right)\beta\left(\tau\right)\;dad\tau\\ &=n_{ct}(t)+n_{cf}(t)\end{aligned}$$

6 $${m}_c(t)=\left(1-{\epsilon}_{ct}\right){\int}_0^{\infty }{\int}_0^{\tau }{n}_{im}\left(t-\tau, t-a\right){F}_{h\prime}\left(\tau \right)\beta \left(\tau \right)\ dad\tau +\left(1-{\epsilon}_{cf}\right){\int}_0^{\infty }{\int}_0^{\tau }{m}_{im}\left(t-\tau, t-a\right)\beta \left(\tau \right)\ dad\tau$$

The first component of both Eqs. (5) and (6) is the community infections generated by notified imported cases while the second component of both equations is the community infections generated by missed imported infectors. The effectiveness of contact tracing in identifying new secondary cases linked to notified cases and the effectiveness of case finding in identifying new cases that are not linked to any existing cases are *ϵ*_*ct*_ and *ϵ*_*cf*_, respectively. *F*_*h*′_(*τ*) is the cumulative probability that an imported case is at large in the community *τ* time since infection and prior to notification (and hence isolation in a hospital or managed facility) (Eq. 7). Using symptomatic cases, we estimate the probability density function of an imported case being isolated *x* time since infection, *f*_*h*′_(*x*), by convolving the incubation period for SARS-CoV-2 infection and the observed time from symptoms onset to isolation of imported cases, *f*_*sh*′_[(*t* − *h*′) − (*t* − *s*)] (Eq. 8). *s* − *h*^′^ is the time delay to developing symptoms since isolation in imported cases and *s* − *h*^′^ > 0 implies that the case was symptomatic before isolation and vice versa.

7 $${F}_{h\prime}\left(\tau \right)=1-{\int}_0^{\tau }{f}_{h\prime }(u) du$$

8 $${f}_{h\prime }(x)={\int}_0^{\infty }{f}_s(u){f}_{sh\prime}\left(x-u\right)\ du$$

Subsequent generations of community infections follow the same principles in Eqs. (5) and (6) as follows in Eqs. (9) and (10). *F*_*h*_(*τ*) is the cumulative probability that a local case is at large in the community *τ* time since infection and prior to notification (and hence isolation in a hospital or managed facility) and derived using the observed time from symptoms onset to isolation in local cases.

9 $$\begin{aligned} {n}_c(t)&={\epsilon}_{ct}{\int}_0^{\infty }{n}_c\left(t-\tau \right){F}_h\left(\tau \right)\beta \left(\tau \right)\ d\tau +{\epsilon}_{cf}{\int}_0^{\infty }{m}_c\left(t-\tau \right)\beta \left(\tau \right)\ d ad\tau \\ &={n}_{ct}(t)+{n}_{cf}(t) \end{aligned}$$

10 $${m}_c(t)=\left(1-{\epsilon}_{ct}\right){\int}_0^{\infty }{n}_c\left(t-\tau \right){F}_h\left(\tau \right)\beta \left(\tau \right)\ d\tau +\left(1-{\epsilon}_{cf}\right){\int}_0^{\infty }{m}_c\left(t-\tau \right)\beta \left(\tau \right)\ d\tau$$

Given the potential for early case isolation at any time point, the reproduction number of a notified community case ${R}_n={\int}_0^{\infty }{F}_h\left(\tau \right)\beta \left(\tau \right) d\tau$is lower than that of a missed case ${R}_m=R={\int}_0^{\infty}\beta \left(\tau \right) d\tau$. Overall, the effective reproduction number in the community, *R*_*eff*_, is an aggregate measure of both *R*_*n*_ and *R*_*m*_ whose value corresponds to the dominant eigenvalue of the next generation matrix, *K*, as follows:

11 $$K=\left[\begin{array}{cc}\left(1-{\epsilon}_{cf}\right){\int}_0^{\infty}\beta \left(\tau \right)\ d\tau & \left(1-{\epsilon}_{ct}\right){\int}_0^{\infty }{F}_h\left(\tau \right)\beta \left(\tau \right)\ d\tau \\ {}{\epsilon}_{cf}{\int}_0^{\infty}\beta \left(\tau \right)\ d\tau & {\epsilon}_{ct}{\int}_0^{\infty }{F}_h\left(\tau \right)\beta \left(\tau \right)\ d\tau \end{array}\right]$$

### Model fitting

We assumed the infection was first introduced into a naïve population by imported cases and disease transmission was simulated over calendar time through a branching process using Eqs. (5) to (10). Early isolation of notified infected individuals and modelled outbreak control measures such as border controls (*ρ*), case finding (*ϵ*_*cf*_), contact tracing (*ϵ*_*ct*_), other non-pharmaceutical interventions (*R*) (Table 1) would influence the trajectory of the notified cases and the expected incidence was fitted using a negative binomial likelihood to the observed daily incidence of linked and unlinked local COVID-19 cases isolated in hospitals or managed facilities (i.e. *i*_*ct*_(*t*) and *i*_*cf*_(*t*)). The modelled linked and unlinked cases isolated at time *t* are defined as:

12 $${h}_{ct}(t)={\int}_0^{\infty }{n}_{ct}\left(t-\tau \right){f}_h\left(\tau \right) d\tau$$

13 $${h}_{cf}(t)={\int}_0^{\infty }{n}_{cf}\left(t-\tau \right){f}_h\left(\tau \right) d\tau$$

We defined the likelihood of observing unlinked and linked cases at the time of isolation as:

14 $${L}_t={P}_{nbinom}\left[\ {i}_{ct}(t)\ |\ {h}_{ct}(t)\right]\times {P}_{nbinom}\left[\ {i}_{cf}(t)\ |\ {h}_{cf}(t)\right]$$

The final likelihood of the community infections over the course of a period of interest is:

15 $$L=\prod\limits_t{L}_t$$

For sensitivity analysis, we assumed that the observed data was not stratified into linked and unlinked cases and the likelihood function was defined as:

16 $${L}_t={P}_{nbinom}\left[\ {i}_c(t)\ |\ {h}_c(t)\right]$$

Using Eqs. (14) and (16), we could estimate the lower and upper limits on the median number of missed infections respectively as the former assumes no misclassification on the source of infection for a case, while the latter tends to exhibit wider uncertainty as it does not account for the source of infection of a locally infected case. In reality, misclassification could occur during cluster investigation and data processing for a large number of cases, but some information on case linkage would exist and lend support to the analysis if contact tracing and testing of exposed contacts was implemented.

Given the long time series of data available for modelling, we subset the wild-type SARS-CoV-2 and Delta variant notified cases a priori, into different time periods in 2020 and 2021 (Table 2). From Apr 24, 2021, onwards, non-residents with a travel history to India were not allowed entry into Singapore or transit through Singapore in response to the surge in Delta variant cases reported in India [33]. This was extended to include Bangladesh, Nepal, Pakistan and Sri Lanka from 2 May onwards [34]. Following the tightening of border controls, the notified COVID-19 cases among travellers from May 16, 2021, onwards reduced to an average of 5 cases per day and the missed imported infections from this date onwards were assumed to be negligible.

**Table 2:** Time periods considered for wild-type SARS-CoV-2 transmission during 2020 and Delta variant transmission during 2021

SARS-CoV-2 lineage	Time period	Description
Wild-type	Jan 18–Feb 29, 2020	Transmission driven by travellers arriving from Wuhan
	Mar 1–Apr 6, 2020	Returning travellers from countries with ongoing outbreaks
	Apr 7–Jun 18, 2020	Increased reopening of national borders
	Jun 19–Jul 12, 2020	Resumption of more local activities
	Jul 13–Dec 31, 2020	Increased reopening of national borders
Delta variant	Apr 1–May 12, 2021	Transmission driven mainly by travellers arriving from India
	May 13–Jun 20, 2021	Tightening of outbreak control measures before relaxation of measures in mid-June
	Jul 1–Jul 17, 2021	Nightclub and fishery port outbreak clusters
	Jul 18–Aug 18, 2021	Tightening of outbreak control measures

Model fitting was performed using a Markov chain Monte Carlo (MCMC) algorithm with an adaptive multivariate normal proposal distribution [35] and the assumed informative priors are listed in Additional file 1: Table S1. Sensitivity analysis was performed assuming uniform priors. Four chains were run with a burn-in of 5000 iterations and samples were thinned every 50 iterations. Convergence was assessed through visual inspection of the Gelman-Rubin convergence statistic and trace plots. The posterior distribution of the parameters in each time period was estimated via MCMC sampling from 23,200 draws.

### Burden of disease and infection

In Singapore, all pneumonia deaths or deaths from unknown causes were subjected to SARS-CoV-2 testing [3, 25]. Hence, the extent of underreporting for SARS-CoV-2 deaths was expected to be low during the study period of interest. The average risk of ICU admission among cases was the proportion of cases admitted into the ICU over all notified cases and the average case fatality ratio was the proportion of deaths among all notified cases. The average risk of ICU admission among all infections and the average infection fatality ratio was also computed using the modelled total infections.

### Comparing outbreak metric between using notified cases only and with inclusion of missed cases

We calculated the proportion of unlinked cases over all notified confirmed cases as this metric is commonly used in the COVID-19 pandemic and in previous outbreaks of other infectious diseases to proxy the extent of missed infections [36–42]. Using the modelled missed and notified infections, we derived the level of case ascertainment (i.e. the proportion of notified cases to the total number of infections) and compared both outbreak metrics. All modelled data were presented as the median with 95% credible intervals (CrI).

### Independent model validation

We validated the model outcomes against an independent, cross-sectional population seroprevalence survey conducted from Sep 7 to 31, 2020, with 1578 participants randomly selected from the general population (Chen MI-C, Lim VWX. Updates on the sero-epidemiology of SARS-CoV-2 in Singapore, and reflections on the role of post-vaccine sero-surveillance, unpublished). Serology was performed using commercially available test kits from Roche, Wondfo and GenScript cPass S Protein RBD Neutralization Antibody Detection Kit, a SARS-CoV-2 surrogate virus neutralisation test (sVNT), a pseudovirus-based VNT (pVNT) and an S protein flow-based assay [43–45]. Accounting for the seroconversion probability and IgG detection probability since time of infection, we estimated the number of serology positive cases and compared them against the seroprevalence rate in the general population as follows:

17 $${\int}_{T_s}^{T_e}{\int}_0^{\infty }{f}_T(t)\left[{n}_c\left(t-\tau \right)+{m}_c\left(t-\tau \right)\right]\ {p}_s\ {f}_p\left(\tau \right)\ d\tau dt$$

where *f*_*T*_(*t*) is a uniform probability distribution of being tested on a day from Sep 7 to Oct 31, 2020 (*T*_*s*_ and *T*_*e*_ inclusive of both dates), *p*_*s*_ is the probability of seroconversion [46], *f*_*p*_(*τ*) is the probability of being detected serology positive *τ* time since infection given seroconversion. We assumed the serology detection probabilities approach 1 after 30 days from time of infection and no decline in immunity was observed up to 11 months post infection [47]. Sensitivity analysis was performed assuming approximately 40% decline in antibody levels 3 months post infection and about 80% decline by 11 months post infection [48, 49]. Observed data were presented as the mean and the 95% confidence intervals (CI) for binomial proportions were computed using Wilson’s method [50]. We bootstrapped the difference between the observed and modelled rates and this difference was considered statistically significant if the 95% CI does not contain zero.

## Results

Combining multiple data streams with a transmission model, we compared the effectiveness of respective outbreak control measures and epidemiological characteristics for different circulating SARS-CoV-2 variants.

### Effectiveness of border control

**Fig. 2:**
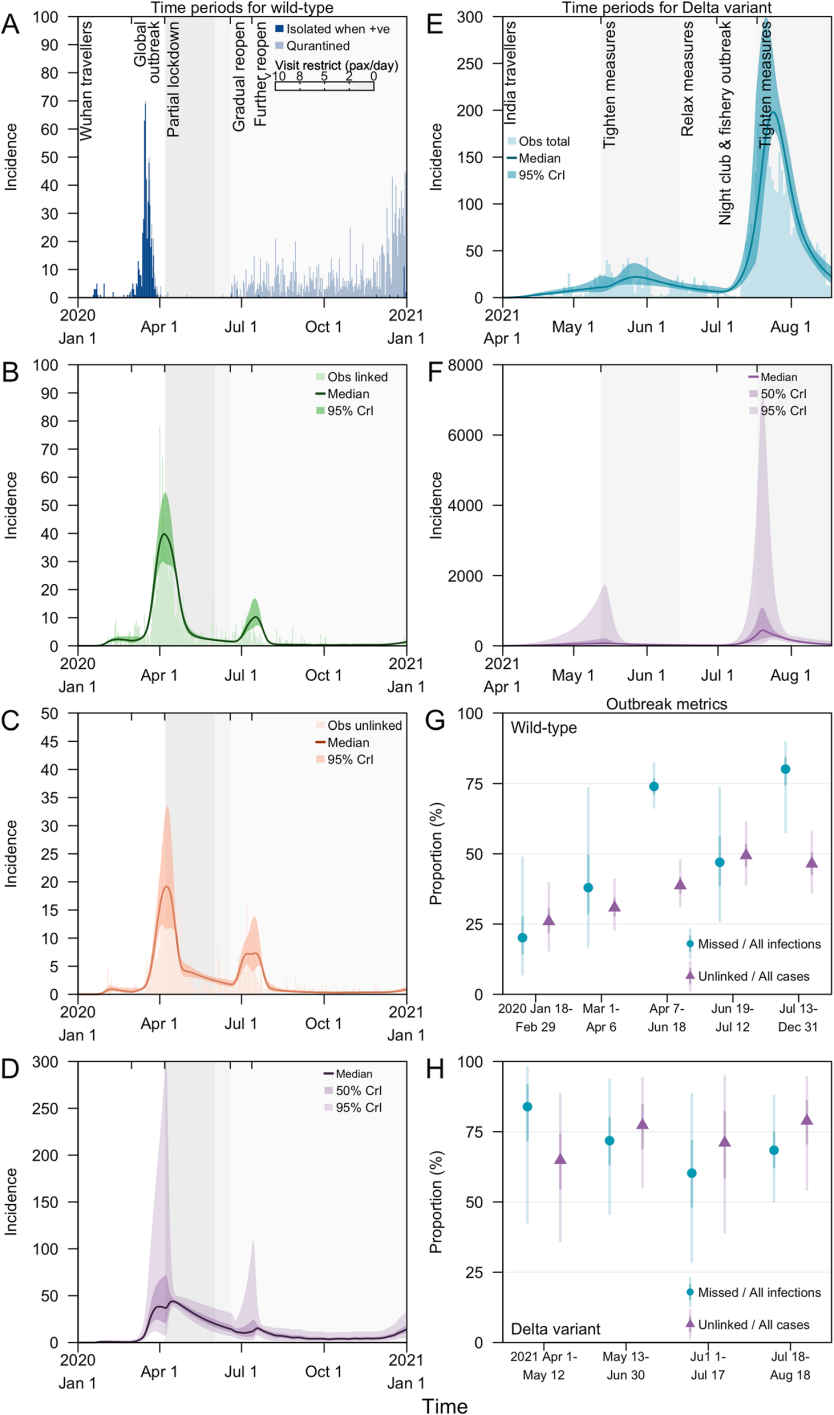
Daily incidence of COVID-19 cases in Singapore arising from wild-type SARS-CoV-2 transmission in 2020, **A** notified imported cases who were isolated after testing positive or quarantined upon arrival, **B** notified local linked cases and modelled posteriors, **C** notified local unlinked cases and modelled posteriors, and **D** modelled posteriors for local missed infections. Daily incidence of COVID-19 cases in Singapore arising from SARS-CoV-2 Delta variant transmission in 2021, **E** notified local cases and modelled posteriors and **F** modelled posteriors for local missed infections. Grey-shaded areas in **A**–**F** represent periods with movement and visitor restrictions with darker shades signifying a reduced number of visitors to each household per day. Modelled posterior outbreak metrics for **G** wild-type SARS-CoV-2 transmission in 2020 and **H** Delta variant transmission in 2021

**Fig. 3:**
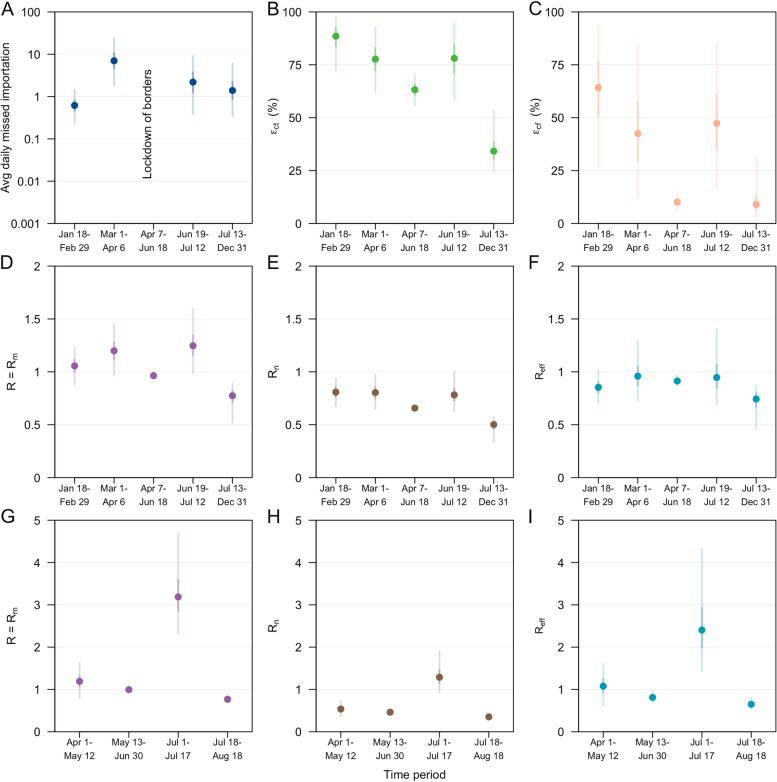
Model parameter estimates on SARS-CoV-2 transmission. **A** average daily missed imported infections in log scale, **B** effectiveness of contact tracing in detecting a linked case, *ϵ*_*ct*_, **C** effectiveness of case finding in detecting an unlinked case, *ϵ*_*cf*_. Reproduction number for wild-type SARS-CoV-2 in 2020 (**D**–**F**) and Delta variant in 2021 (**G**–**I**). **D**, **G ***R* or *R*_*m*_, of a missed COVID-19 case, **E**, **H ***R*_*n*_, of a notified COVID-19 case, and **F**, **I** effective reproduction number, *R*_*eff*_. Estimates of the posterior median (dot), 50% CrI (dark vertical lines) and 95% CrI (light vertical lines) as shown

The earliest measure implemented to minimise the introduction of wild-type SARS-CoV-2, and later also used to delay the Delta variant, was border control measures. Initial measures from Jan 18 to Feb 29, 2020, aimed to reduce the spread of SARS-CoV-2 by infected persons arriving from China. While there was less than 1 notified imported case per day during this period (Fig. 2A), we estimated that there were 0.6 missed imported infections per day (95% credible intervals, CrI 0.2–1) (Fig. 3A) or equivalent to 0.9 missed imported infections per notified case (95% CrI 0.4–2) (Additional file 1: Table S2). From Mar 1 to Apr 6, 2020, there was a surge of 15 notified imported cases per day returning from other countries with ongoing outbreaks (Fig. 2A) and we estimated a median of 7 missed imported infections per day (95% CrI 2–24) (Fig. 3A) or 0.5 missed imported infections per notified imported case (95% CrI 0.1–2) (Additional file 1: Table S2). During this period, border control measures were tightened and incoming travellers were progressively subjected to quarantine in managed institutions. Despite the decline in notified imported cases from Mar 16 to Apr 1, 2020, persistent community transmission prompted a nationwide partial lockdown on Apr 7, 2020 (Fig. 2A–C) where non-essential workers were required to work from home, students transited to home-based learning and non-essential facilities and services ceased operations [51].

Following the partial lockdown, the reopening of borders and hence the risk of disease introduction was carefully balanced against the resumption of community activities and the associated risk of community transmission. From Jul 13 to Dec 31, 2020, there were 7 notified cases per 1,000 travellers, three times higher than the period prior to lockdown (i.e. 2 notified cases per 1,000 travellers from Mar 1 to Apr 6, 2020) but the number of imported cases who were not quarantined upon arrival was kept low at less than 0.1 cases per 1000 arrivals. Furthermore, with the strict quarantine of incoming travellers and continued enforcement of outbreak control measures, the average daily number of missed imported infections declined to 2 cases (95% CrI 0.3–6) from Jul 13 to Dec 31, 2020 (Fig. 3A) or 0.2 missed imported infections per notified imported case (95% Crl 0.04–0.7) (Additional file 1: Table S2) with no signs of a growing outbreak (Fig. 2A–C).

From Apr 1 to May 12, 2021, while the country continued to enforce quarantine for the majority of the incoming travellers in managed institutions, there was an average of 14 notified cases per 1000 travellers during this period. This was the highest level in our study window. The surge was attributed to imported cases with travel from India. While notified community COVID-19 cases from Apr 1 to May 12, 2021, were low with an average of six cases per day, the occurrence of increased transmission and COVID-19 clusters at the international airport prompted the tightening of COVID-19 outbreak control measures [46]. Despite imposing a travel ban to all non-residents with a travel history to India from Apr 23, 2021, onwards [27], we estimated 4 missed imported infections per day (95% CrI 1–26) or 0.3 missed imported infections per notified imported case (95% CrI 0.05–1.3) (Additional file 1: Table S2).

### Effectiveness of case finding and contact tracing

We estimated the country’s contact tracing system was able to detect over 78% of the secondary infections generated by notified cases ( *ϵ*_*ct*_, 95% CrI 62–93%) from Mar 1 to Apr 6, 2020 (Fig. 3B). However, the effectiveness of case finding which depends on the overall testing capacity, the extent of surveillance and the health-seeking behaviour of the population was at 64% (*ϵ*_*cf*_, 95% CrI 27–93%) during the start of the outbreak and declined to 42% (*ϵ*_*cf*_, 95% CrI 12–84%) prior to the partial lockdown in Apr 2020 (Fig. 3C). One week before the partial lockdown, there were an average of 16 unlinked cases per day and we estimated 120 missed infections (95% CrI 25–870) per day signifying substantial gaps in the transmission chains. Consequently, *ϵ*_*ct*_ and *ϵ*_*cf*_ during the lockdown was lowered to 63% (95% CrI 56–71%) and 10% (95% CrI 7–15%), respectively (Fig. 3B and C).

As social and economic activities progressively resumed from Jun 19, 2020, onwards, we estimated an increase in *ϵ*_*ct*_ to 78% (95% CrI 58–94%) and *ϵ*_*cf*_ to 47% (95% CrI 17–85%) (Fig. 3B and C). This finding is in line with the broadening of the close contact definition, use of contact tracing devices to facilitate contact tracing, implementation of large-scale swab operations to limit spread from outbreak clusters and increased use of rapid antigen tests for routine surveillance in targeted groups [52–55].

Across all time periods in 2020, *ϵ*_*cf*_ exhibits wide credible intervals as a result of some correlation with the factor, *ρ*, which scales the extent of missed imported infections (Additional file 1: Fig. S1). Similar estimates of *ϵ*_*ct*_ and *ϵ*_*cf*_ were obtained when using uniform priors for sensitivity analysis (Additional file 1: Figs. S2 and S3).

### Community reproduction number

Prior to the partial lockdown, the average number of secondary cases generated by a single infectious individual, *R*, was estimated to be 1.2 cases (95% CrI 1.0–1.4) from Mar 1 to Apr 6, 2020, but the observed reproduction number among chains of notified cases was lower at 0.8 cases (*R*_*n*_, 95% CrI 0.7–1.0) due to the reduced amount of time spent in the community while infectious compared to a missed infection (Fig. 3D and E). Overall, *R*_*eff*_ was 1.0 cases (95% CrI 0.7–1.3) resulting in a sustained cumulative increase of cases (Fig. 3F). During the partial lockdown in 2020, we estimated *R*_*eff*_ to be below 1 at 0.9 cases (95% CrI 0.9–1.0). While this signalled a declining outbreak, it took approximately one month to reach a daily incidence of less than 10 cases (Fig. 2B and C).

From Jan 18 to Jun 18, 2020, the daily number of notified cases in the community was at least 10 cases per day. Using a model fitted against notified cases without stratifying the data into linked and unlinked cases for sensitivity analysis, the median estimates for *R* were similar to the above main analysis, although wider uncertainty intervals were observed due to the lack of information on case linkage to constraint estimates (Additional file 1: Fig. S4).

Outbreak control measures were tightened from May 16, 2021, onwards and the average daily COVID-19 Delta variant community cases declined to less than 10 from Jun 14 to Jul 30, 2021, with a *R* of 1.0 cases (95% CrI 0.9–1.1) (Fig. 3G). However, a rapid increase of COVID-19 cases was observed in Jul 2021 and epidemiological investigations pointed to transmissions at nightclubs and at a fishery port [49]. This rapid growth was made possible when *R* was approximately 3.2 cases (95% CrI 2.3–4.7) but model fitting suggested that this lasted for about 2 weeks from Jul 1 to 17, 2021 (Figs. 2E and 3G). With extensive testing and clamp down of underground nightclubs following detection on Jul 12, 2021, cases were progressively notified over the following week and showed signs of decline prior to the tightening of control measures on Jul 22, 2021. When adjusting for the effect of varying vaccination, the reproduction number across the time periods of study in the Delta variant outbreak was scaled up by 1.2–1.5 times (Additional file 1: Fig. S5). The reproduction number represented the risk arising from other population interventions or human behaviour, in the absence of vaccination and was above 1 as the country progressively reopened and relaxed the outbreak restrictions following an increase in vaccination coverage.

When using a uniform prior for analysis, model fitting for the Delta variant outbreak showed similar outputs to the case incidence and reproduction number from Apr 1 to May 12, 2021, and May 13 to Jun 30, 2021 (Additional file 1: Figs. S6 and S7). However, outputs for the uniform prior diverge from the observed data and the outputs of the informative prior for Jul 1 to Jul 17, 2021, and Jul 18 to Aug 18, 2021—this deviation will be addressed further in the ‘Discussion’ section.

### Burden of disease and infection

Using the incidence of both linked and unlinked cases, our main analysis estimated 730 missed infections (95% CrI 230–3600) (Table 3 and Fig. 2D) from Mar 1 to Apr 6, 2020, which translates to approximately 20 missed infections per day (95% CrI 6–96). During the partial lockdown period and the succeeding period (Apr 7–Jun 18, 2020), the number of missed infections per day decreased to 30 (95% CrI 20–56). As border restrictions were gradually lifted and economic and social activities resumed from Jun 19, 2020, onwards, the daily missed infections remained low at 7 infections (95% CrI 3–20). Overall, we estimated that 4,400 infections (95% CrI 2400–11,000) were missed in 2020 or equivalent to 63% of all infections (95% CrI 49–78%) (Table 3 and Fig. 2D).

**Table 3:** Summary of observed data and modelled outputs (median and 95% CrI in parenthesis) by respective time periods in 2020 for wild-type SARS-CoV-2 transmission

Observed data (●) and modelled outputs (◆)	Time period in 2020					
	Overall Jan–Dec	Jan 18–Feb 29	Mar 1–Apr 6	Apr 7–Jun 18	Jun 19–Jul 12	Jul 13–Dec 31
● Imported cases						
Isolated for testing on arrival or quarantined	1653	0	50	5	78	1520
Not quarantined	547	29	497	0	4	17
● Local cases (by time of isolation)						
Linked	1505	65	606	610	113	111
Unlinked	864	20	204	420	107	113
◆ Missed cases	4400 (2400–11,000)	25 (8–100)	730 (230–3600)	2200 (1500–4100)	280 (100–1100)	1100 (360–2800)
◆ Total cases (adjusted by time of infection and missed cases)	7100 (4800–14,000)	130 (90–220)	1900 (1300–4900)	2900 (2200–5100)	590 (350–1500)	1400 (620–3100)
● ICU cases (by time of isolation)	86	13	44	28	1	0
● Deaths (by time of isolation)	22	2	11	9	0	0
◆ Case ICU risk (%)	3.3 (2.5–4.0)	23.2 (16.2–32.4)	4.0 (3.0–5.0)	2.0 (1.5–2.4)	0.3 (0.2–0.4)	0 (0–0)
◆ Infection ICU risk (%)	1.2 (0.6–1.8)	18.2 (10.8–26.3)	2.4 (1.0–3.7)	0.5 (0.3–0.7)	0.2 (0.07–0.3)	0 (0–0)
◆ Case fatality ratio (%)	0.8 (0.6–1.0)	3.8 (2.6–5.3)	1.2 (0.9–1.5)	0.5 (0.4–0.6)	0 (0–0)	0 (0–0)
◆ Infection fatality ratio (%)	0.3 (0.2–0.5)	3.0 (1.7–4.3)	0.7 (0.3–1.1)	0.1 (0.07–0.2)	0 (0–0)	0 (0–0)

Our preceding main analysis incorporated the additional case linkage information provided by case finding and contact tracing (i.e. linked and unlinked cases). When model fitting during sensitivity analysis was performed using the time series of all notified cases without stratification by case linkage, we estimated approximately 1900 infections (95% CrI 600–10,000) were missed prior to the partial lockdown in Apr 2020 or 50 missed infections per day (95% CrI 10–280) (Additional file 1: Table S3). Contrary to the previous model fit, we estimated approximately 130 missed infections per day (95% CrI 80–300) during the partial lockdown, and this was approximately 4 times (95% Crl 4–5) higher than of the previous model fit (Additional file 1: Table S3). We estimated that 15,000 infections (95% CrI 8,400–38,000) were missed in 2020 (Additional file 1: Table S3 and Fig. S8).

Both the main and sensitivity analysis for the wild-type SARS-CoV-2 serve as the lower and upper limit of the modelled missed infections. The former assumed perfect classification of case linkages while the latter was derived without using the case linkage information to constrain the range of parameters that reproduces the modelled outbreak, resulting in wider uncertainty intervals in the estimated missed infections.

From Apr 1 to May 12, 2021, more than 60% of the cases and more than 65% of the population were unvaccinated. Singapore experienced a surge in notified imported cases and consequently missed imported infections. Using all notified Delta variant cases without stratification by case linkage, we estimated that 1,400 community infections were missed (95% CrI 200–15,000) during this period (Table 4 and Fig. 2F). Rapid transmission arising from nightclub clusters and a fishery port followed by extensive case finding measures such as large-scale swab operations resulted in 80 missed infections per day (95% CrI 16–700) from Jul 18 to Aug 18, 2021. Despite the shorter study period for Delta variant transmission as compared to the wild-type SARS-CoV-2, we estimated that there were 12,000 missed infections (95% CrI 4200–75,000), or equivalent to 70% of all infections (95% CrI 49–91%), in a span of about 5 months.

**Table 4:** Summary of observed data and modelled outputs (median and 95% CrI in parenthesis) by respective time periods in 2021 for SARS-CoV-2 Delta variant transmission

Observed data (●) and modelled outputs (◆)	Time period in 2021				
	Overall Apr–Aug	Apr 1–May 12	May 13–Jun 30	Jul 1–Jul 17	Jul 18–Aug 18
● Imported cases					
Isolated for testing on arrival or quarantined	1291	809	270	136	76
Not quarantined	93	34	32	12	15
● Local cases (by time of isolation	4371	196	755	474	2946
◆ Missed cases	12,000 (4200–75,000)	1400 (180–15,000)	1700 (700–11,000)	1400 (270–11,000)	6100 (2600–43,000)
◆ Total cases (adjusted by time of infection and missed cases)	17,000 (8000–84,000)	1700 (420–15,500)	2400 (1500–12,000)	2400 (800–13,000)	9000 (4700–50,000)
● ICU cases (by time of isolation)	36	3	11	3	19
● Deaths (by time of isolation)	25	3	4	1	17
◆ Case ICU risk (%)	0.7 (0.3–1.1)	1.7 (1.0–2.6)	1.3 (1.0–1.8)	0.8 (0.3–1.7)	0.5 (0.2–0.8)
◆ Infection ICU risk (%)	0.2 (0.04–0.4)	0.3 (0.03–1.1)	0.4 (0.08–0.6)	0.3 (0.06–1.0)	0.2 (0.03–0.3)
◆ Case fatality ratio (%)	0.5 (0.2–0.8)	1.4 (0.8–2.0)	0.6 (0.4–0.8)	0.2 (0.09–0.4)	0.5 (0.2–0.8)
◆ Infection fatality ratio (%)	0.2 (0.03–0.3)	0.2 (0.02–0.9)	0.2 (0.03–0.3)	0.08 (0.01–0.2)	0.2 (0.03–0.3)

Overall, the estimated case fatality ratio was 0.8% (95% CrI 0.6–1.0%) in 2020 and 0.5 (95% CrI 0.2–0.8%) in Apr–Aug 2021, and remains below 1% as of Nov 2021 (Tables 3 and 4). The infection fatality ratio was 0.3% (95% CrI 0.2–0.5%) in 2020 for wild-type SARS-CoV-2 infections and 0.2% (95% CrI 0.033–0.3%) in 2021 for Delta variant infections. The risk of ICU admission among cases was 3.3% (95% CrI 2.5–4.0%) in 2020 and 0.7% (95% CrI 0.3–1.1%) in 2021 and but these estimates were approximately 3 times higher than the risk of ICU admission among infections at 1.2% (95% CrI 0.6–1.8%) in 2020 and 0.2% (95% CrI 0.04–0.4%) in 2021.

### Comparing outbreak metric between using notified cases only and with inclusion of missed cases

When the effectiveness of detecting linked and unlinked cases declined in March 2020 during the surge of imported cases and further declined during the partial lockdown (Fig. 3B and C), we estimated the proportion of missed infections among all infections increased to 74% (95% CrI 67–82%) (Fig. 2G) between Apr 7 to Jun 18, 2020. This was 1.9 times (95% CrI 1.6–2.3) higher than the proportion of cases that was unlinked at 39% (95% CrI 32–48). The proportion of missed infections among all infections was also 1.3 times (95% CrI 0.7–2.3) higher than the proportion of unlinked cases to all cases from Apr 1 to May 12, 2021, when the Delta variant was the predominant circulating strain (Fig. 2H).

During periods of increased testing during reopening, the estimated proportion of missed cases was low at 47% (95% CrI 26–73%) from Jun 19 to Jul 12, 2020; 0.95 times (95% CrI 0.5–1.6) lower that the proportion of cases that was unlinked which was 49% (95% CrI 38–61%) (Fig. 2G). Similarly, from Jul 12 to Aug 18, 2021, where extensive testing was conducted as part of cluster outbreak investigations, we estimated that 68% of all infections were missed (95% CrI 50–88%) and 0.9 times (95% CrI 0.6–1.3) lower than the proportion of cases that was unlinked at 79% (95% CrI 54–94%) (Fig. 2H).

### Independent validation of estimates

While the transmission model was able to reproduce the observed temporal trends, we sought to further validate the model outputs against an independent population-level cross-sectional seroprevalence survey. From Sep 7 to 31, 2020, SARS-CoV-2 antibodies were detected in two out of 1578 participants when subjected to all serological test methods and these participants were also negative for SARS-CoV-1 infection [34]. This translates to an observed seroprevalence of 0.13% (95% confidence intervals, CI 0.03–0.46%). Four other participants had SARS-CoV-2 antibodies detected when twice analysed by the cPass test kit but tested negative on the other serological tests.

Using the linked and unlinked cases in 2020, our model estimates implied a population seroprevalence of 0.05% (95% CI 0.03–0.1%) when assuming no waning immunity up to 11 months after symptoms post infection. When using the notified cases without accounting for their case linkages in 2020 for model fitting in sensitivity analysis, the estimated seroprevalence was revised upwards to 0.13% (95% CI 0.08–0.3%). Both model outcomes were not statistically significantly different from the observed seroprevalence. However, when assuming waning seropositivity 3 months after symptoms onset, the estimated seroprevalence in both models was 0.03% (95% CI 0.02–0.06%) and 0.08% (95% CI 0.05–0.18%).

## Discussion

Using the growth patterns in the daily incidence of local linked and unlinked cases, and imported cases with community contact, we reconstructed the incidence of missed infections over time in Singapore. This enabled us to disentangle the effects of targeted measures such as case finding and contact tracing from other population-wide outbreak interventions. Our modelling framework was able to infer these missed infections without requiring large-scale serological surveys, which are typically challenging to conduct at the start of a pandemic. Such analysis can therefore provide early insights into the effectiveness of respective categories of outbreak control measures, and hence further inform the extent of measures required during different stages of an outbreak.

The changes in the estimated effectiveness of control measures largely coincide with the shifts in outbreak control policies, but there were other likely contributing factors. Changes in human behaviours such as a reduction in health-seeking behaviour coincided with a decline in the effectiveness of case finding, *ϵ*_*cf*_, from 42% in Mar 1 to Apr 6, 2020, to 10% during the lockdown from Apr 7 to Jun 18, 2020 [23]. Furthermore, the interdependence of outbreak control measures can cause the effectiveness of measures to change in tandem. In particular, the contribution of contact tracing towards outbreak control hinges on the extent of case finding. Following the decline in *ϵ*_*cf*_ during the lockdown, the effectiveness of contact tracing in identifying new cases declined from 78% in Mar 1 to Apr 6, 2020, to 63% during the lockdown. This observation is also supported by theory—when the effectiveness of isolating cases is low, a slight decrease in the effectiveness of contact tracing can result in a growing outbreak [30]. Collectively, about 75% of the infections were estimated to be missed during the lockdown and this proportion was higher than other time periods due to the lowered effectiveness in both case finding and contact tracing. Thus, by identifying which outbreak control measures were contributing to the growth of an outbreak and the corresponding reasons for its lowered effectiveness, it is possible to address relevant aspects of human behaviour (e.g. promote use of telemedicine as patients feel more comfortable seeing their doctors online [56]; discourage clinic hopping so the same doctor can better assess the need for follow up test [57]).

In both wild-type SARS-CoV-2 and Delta variant outbreaks in Singapore, on average, there was less than 1 death per day. With prolonged periods of low death counts, we reconstructed the underlying outbreak dynamics using the incidence of linked and unlinked cases instead of using reported fatalities [21, 58]. Prior to 2021, the Singapore population was largely unvaccinated and during the Delta variant outbreak about 60% of the population was vaccinated by Aug 2021. Our CFR estimates were less than 1% for the wild-type SARS-CoV-2 and Delta variant outbreak, which was less than the early CFR estimates of around 1.4% for wild-type SARS-CoV-2 and 3 times lower than the CFR estimates for the Delta variant in other studies [59, 60]. The IFR estimates for both outbreaks in Singapore were also less than 0.5%, and in the lower range of IFR estimates as compared to other countries and regions [58, 61, 62]. While the healthcare system was stretched in both outbreaks, ICU capacity was not exceeded and this helped to keep the number of deaths to a minimum. As deaths observed in small outbreak clusters would not be reflective of the number of deaths that could arise during a large epidemic wave, care is needed in the interpretation of underlying infection dynamics and how these influence measured disease outcomes.

We found that metrics derived from observed data alone do not always accurately reflect the underlying outbreak. Specifically, metrics such as the proportion of unlinked cases among all notified cases are not necessarily representative of the proportion of missed infections among all infections, and policy makers should therefore be careful when drawing conclusions of the latter from the former. This discrepancy is likely to occur because the missed infections have a much higher reproduction number as compared to notified cases, or when a single missed infection is the source of infection for multiple unlinked cases and the outbreak could be misinterpreted as growing or declining slowly in either scenario. In contrast, contact tracing data provides additional information on the source of infection of a case. The collection of such data expends minimal effort yet can help to improve our understanding of the underlying outbreak although misclassification could also affect the interpretation of the outbreak dynamics. Thus, the interpretation of common metrics should be done with a clear understanding of the data collection process. Previous studies have estimated the impact of measures such as border control by assessing correlations between the timing of interventions and national-level case incidence [63], but our results suggest such analysis will not capture the complexity of interacting measures against a background of changing infection detection.

We also found that multiple independent notification datasets and informative priors helped to disentangle the model parameters and achieve more precision in estimates. Unlinked cases were generated by either missed imported or local infections with the former modelled as a factor of the notified imported cases, *ρ*. As such, the interaction of model parameters results in wide 95% credible intervals for *ϵ*_*cf*_ estimates. To improve these estimates, we could further stratify exposure histories of unlinked cases by their interactions with travellers from countries with ongoing outbreak for model fitting. Informative and uniform priors produced a similar set of parameter estimates when there were multiple independent notification data in the SARS-CoV-2 wild-type outbreak in 2020 for model fitting. However, the model output using a uniform prior was different from that of an informative prior for the Delta variant outbreak in Jul to Aug 2021. Unlike the wild-type SARS-CoV-2 outbreak, model fitting for the Delta variant was based on the time series of cases without accounting for the case linkage. As such, there was limited data to inform the extent of underreporting and hence the number of missed infections. The estimates of *R* from Jul 1 to 17, 2021 when using the informative prior falls within the lower range of the estimates derived from the uniform prior although both analyses suggest a growing outbreak.

There are some additional limitations to our study. One is that asymptomatic cases were assumed to have a similar distribution of delay from the time of infection to notification as symptomatic cases. To circumvent this, we can study the changes in the trajectory of the cycle threshold values (proxy for viral load) of cases that were tested multiple times over the course of the infection. The infection time of symptomatic and asymptomatic cases can be estimated from their respective viral growth trajectory [64, 65] thereby informing the delay distribution for respective types of cases. Furthermore, we assumed that asymptomatic cases were as infectious as symptomatic individuals, and hence, no stratification of *R* was modelled as there is no strong evidence to suggest that asymptomatic SARS-CoV-2 infections are less infectious than symptomatic individuals [66, 67]. Our modelled outcomes for wild-type SARS-CoV-2 transmission were able to reproduce independent observations in a separate population-level serological survey and this lends support to our assumption of a homogeneous *R* among most missed infections.

In addition, the burden of disease and infection estimates were averaged across all age groups, as there was insufficient data to estimate the transmissibility and susceptibility across different age groups in each time period. In our branching process model, we also assumed that each of the four parameters remains constant in a specified time period. As such, we are unable to provide a time-varying measure to characterise the impact of different outbreak detection and control measures that were progressively rolled out in the population at a granular level. Instead, time periods were chosen based on prior knowledge of major policies that would affect at least one of the four model parameters. In particular, from Jul 1 to 17, 2021, the outbreak of COVID-19 cases from a nightclub cluster and fishery port resulted in a reproduction number of more than 1. For cases at the end of this time period, the model assumes that their *R* is the same as the cases at the start of the same time period. However, as rapid and strict outbreak control measures were implemented around the period of Jul 18, 2021, the *R* of the cases around this transition period is expected to vary between the reproduction number estimated for Jul 1 to 17, 2021 and Jul 18 to Aug 18, 2021. With the potential for a larger reproduction number using a uniform prior, the exponential number of new infections generated by cases around the transition period causes the modelled peak outbreak to overshoot the observed peak in the subsequent time period. This further highlights the importance of having multiple independent data on case linkage to better inform the parameter estimates and to infer missed infections.

## Conclusions

The SARS-CoV-2 pandemic has generated many new and expanded data streams alongside new ways to reconstruct outbreak dynamics and evaluate the extent of missed infections, even in the presence of high asymptomatic rates and underreporting of cases. Our results show that data on case linkage can help countries evaluate their performance in case finding, contact tracing and the effectiveness of their border restrictions. Relying simply on the interaction of missed and notified infections can introduce unseen heterogeneity into the reproduction number and hence create a false picture of a controlled outbreak. As countries deal with future waves of COVID-19 or plan for pandemics in the future, it will be important to have an integrated surveillance and modelling analysis system that can estimate these crucial undetected transmission events.

## Supplementary Information

**Additional file 1: Table S1.** Mathematical notations. **Table S2.** Notified and modelled missed imported wild-type SARS-CoV-2 infections in 2020. **Table S3.** Summary of observed data and modelled outputs for wild-type SARS-CoV-2 transmission. **Fig. S1.** Contour plots to show the correlation between model parameters. **Fig. S2.** Posterior estimates for model fitted to time series of linked and unlinked SARS-CoV-2 wild type cases in 2020 using informative and non-informative priors. **Fig. S3.** Posterior density of the parameters for model fitted to time series of linked and unlinked SARS-CoV-2 wild type cases in 2020 using informative and non-informative priors. **Fig. S4.** Reproduction number, R of a SARS-CoV-2 wild-type in 2020 and Delta variant case in 2021. **Fig. S5.** Reproduction number, R of a SARS-CoV-2 Delta variant case in 2021 after adjusting for vaccine coverage and vaccine effectiveness and using notified cases with no information of the case linkage for model fitting. **Fig. S6.** Posterior estimates for model fitted to time series of SARS-CoV-2 Delta variant cases (without accounting for case linkage) in 2021 using informative and non-informative priors. **Fig. S7.** Posterior density of the parameters for model fitted to time series of linked and unlinked SARS-CoV-2 Delta variant cases (without accounting for case linkage) in 2021 using informative and non-informative priors. **Fig. S8.** Daily incidence of COVID-19 cases in Singapore arising from SARS-CoV-2 wild-type transmission in 2020. **Fig. S9.** Markov chain Monte Carlo trace plots for parameters modelling wild-type SARS-CoV-2 transmission in 2020. **Fig. S10.** Markov chain Monte Carlo trace plots for parameters modelling SARS-CoV-2 Delta variant transmission in 2021.

## Data Availability

All data are available in the manuscript or the supplementary information. The data and code used for our analyses are publicly available at 10.5281/zenodo.7538047.
