# Supplementary material for: Relative role of border restrictions, case finding and contact tracing in controlling SARS-CoV-2 in the presence of undetected transmission: a mathematical modelling study
Source: BMC Med. 2023 Mar 16;21:97. doi: 10.1186/s12916-023-02802-0 (PMC10019421; doi:10.1186/s12916-023-02802-0)
Supplement: Supplementary file 1 — Additional file 1: Table S1. Mathematical notations. Table S2. Notified and modelled missed imported wild-type SARS-CoV-2 infections in 2020. Table S3. Summary of observed data and modelled outputs for wild-type SARS-CoV-2 transmission. Fig. S1. Contour plots to show the correlation between model parameters. Fig. S2. Posterior estimates for model fitted to time series of linked and unlinked SARS-CoV-2 wild type cases in 2020 using informative and non-informative priors. Fig. S3. Posterior density of the parameters for model fitted to time series of linked and unlinked SARS-CoV-2 wild type cases in 2020 using informative and non-informative priors. Fig. S4. Reproduction number, R of a SARS-CoV-2 wild-type in 2020 and Delta variant case in 2021. Fig. S5. Reproduction number, R of a SARS-CoV-2 Delta variant case in 2021 after adjusting for vaccine coverage and vaccine effectiveness and using notified cases with no information of the case linkage for model fitting. Fig. S6. Posterior estimates for model fitted to time series of SARS-CoV-2 Delta variant cases (without accounting for case linkage) in 2021 using informative and non-informative priors. Fig. S7. Posterior density of the parameters for model fitted to time series of linked and unlinked SARS-CoV-2 Delta variant cases (without accounting for case linkage) in 2021 using informative and non-informative priors. Fig. S8. Daily incidence of COVID-19 cases in Singapore arising from SARS-CoV-2 wild-type transmission in 2020. Fig. S9. Markov chain Monte Carlo trace plots for parameters modelling wild-type SARS-CoV-2 transmission in 2020. Fig. S10. Markov chain Monte Carlo trace plots for parameters modelling SARS-CoV-2 Delta variant transmission in 2021. [file 12916_2023_2802_MOESM1_ESM.docx]

**Additional File 1: Relative role of border restrictions, case finding and contact tracing in control SARS-CoV-2 in the presence of undetected transmission: a mathematical modelling study**

Rachael Pung^1,2^*, Hannah E. Clapham^3^, Timothy W. Russell^2^, CMMID COVID-19 Working Group^2^, Vernon J. Lee^1,3^**, Adam J. Kucharski^2^**

^1^Ministry of Health, Singapore

^2^Centre for the Mathematical Modelling of Infectious Diseases, London School of Hygiene and Tropical Medicine

^3^Saw Swee Hock School of Public Health, National University of Singapore, Singapore

*For correspondence [rachael.pung@lshtm.ac.uk](mailto:rachael.pung@lshtm.ac.uk)

**These authors contributed equally to this work.

**Table S1 Mathematical notations**

| Category | Notation | Description |
| --- | --- | --- |
| Time | $\tau$ or $x$ | Time since infection |
|  | $t$ | Calendar time |
|  | $a$ | Duration since arrival; $t-a$ is time of arrival |
|  | $s$ | Duration since symptoms onset; $t-s$ is the time of symptoms onset |
|  | $T_{s} / T_{e}$ | Start / end time of cross-sectional population seroprevalence survey from Sep 7 to Oct 31, 2020 |
| Observed incidence | $i_{cf}\left( t \right)$ | Unlinked cases isolated at time $t$ |
|  | $i_{ct}\left( t \right)$ | Linked cases isolated at time $t$ |
| Probability | $\omega(\tau)$ | Probability density function (PDF) of the time from infection in one case to another (i.e. generation interval). Approximated by the serial interval (i.e. time from symptom onset in one case to another). Modelled as a lognormal distribution with mean 5.9 days and standard deviation 2.4 days [1–3]. |
|  | $f_{a}\left( \tau\right)$ | PDF of arriving to a country $\tau$ time since infection, derived from the convolution of $f_{s}\left( \tau\right)$ and $f_{sa}\left( s-a \right)$ |
|  | $f_{s}\left( \tau\right)$ | PDF of symptom onset $\tau$ time since infection (i.e. incubation period). Modelled as a lognormal distribution with mean 5.8 days and standard deviation 3.1 days for wild-type SARS-CoV-2 [4] and mean 4 days and standard deviation 0.4 days for Delta variant [5]***.*** |
|  | $f_{sa}\left( s-a \right)$ | PDF of time from arrival to symptoms onset using observed data from symptomatic imported cases. |
|  | $f_{h^{'}}(\tau)$ | PDF of an imported case being isolated $\tau$ time since infection, derived from the convolution of $f_{s}\left( \tau\right)$ and $f_{sh^{'}}\left( s-h^{'} \right).$ $f_{h}(\tau)$ for local cases. |
|  | $f_{sh^{'}}\left( s-h^{'} \right)$ | PDF of time from symptoms onset to isolation in an imported case using observed data from symptomatic imported cases. $f_{sh}\left( s-h \right)$ for local cases. |
|  | $F_{h^{'}}\left( \tau\right)$ | Cumulative probability that an imported case is at large in the community $\tau$ time since infection and prior to notification. $F_{h}\left( \tau\right)$ for local cases. |
|  | $f_{p}(\tau)$ | PDF of being seropositive $\tau$ time since infection given seroconversion. Assumed serology detection probabilities approach 1 after 30 days from time of infection and no decline in immunity up to 11 months post infection [6]***.*** Sensitivity analysis was performed assuming approximately 40% decline in antibody levels 3 months post infection and about 80% decline by 11 months post infection [7, 8]. |
|  | $f_{T}\left( t \right)$ | Uniform PDF of being tested on a day between $T_{s}$ and $T_{e}$ (both days inclusive) |
|  | $p_{s}$ | Probability of seroconversion = 0.87 [9]. |
|  | $P_{nbinom}$ | PDF for negative binomial distribution |
| Category | Notation | Description |
| Unknown parameters to be modelled | $\rho$ | Scale parameter to model the missed imported infections as a factor of the notified imported cases  Lognormal(1,1) prior assumed (i.e. the number of missed imported infections is not more than 10 times of notified imported cases in 90% of the time but higher proportions of missed infections is still possible under this sampling framework). |
|  | $\epsilon_{cf}$ | Effectiveness of case finding  Beta(3.1,3.1) prior assumed (i.e. effectiveness of case finding lies in the range of 10-90% for about 99% of the time to prevent sampler from being stuck at the tail ends of the probability range) |
|  | $\epsilon_{ct}$ | Effectiveness of contact tracing  Beta(3.1,3.1) prior assumed (i.e. effectiveness of contact tracing lies in the range of 10-90% for about 99% of the time to prevent sampler from being stuck at the tail ends of the probability range) |
|  | $R$ | Reproduction number or the average number of secondary cases generated by a single infectious individual over the course of the entire infectious period (i.e. no truncation of the infectious period due to quarantine or isolation). Analogous to the reproduction number of a missed infection, $R_{m}$.  Lognormal(1,0.5) prior assumed (i.e. reproduction number is not more than 5 in 90% of the time but higher values are still possible under this sampling framework). |
| Derived parameters | $\beta(\tau)$ | Mean rate at which an infected person infects others (i.e. infectiousness) $\tau$ time since infection |
|  | $R_{n}$ | Reproduction number of a notified case; $R_{n}\leq R_{m}$ |
|  | $R_{eff}$ | Effective reproduction number; $R_{n}\leq R_{eff} \leq R_{m}$ |
|  | $K$ | Next-generation matrix |
|  | $L$ | Likelihood function |
| Modelled incidence | $n_{im}(t) / m_{im}(t)$ | Notified / Missed imported infections infected at time $t$ |
|  | $n_{c}(t) / m_{c}(t)$ | Notified / Missed local infections infected at time $t$ |
|  | $n_{cf}\left( t \right)$ | Unlinked cases infected at time $t$ (i.e. notified local cases with unknown sources of infection) |
|  | $n_{ct}\left( t \right)$ | Linked cases infected at time $t$ (i.e. notified local cases with known sources of infection) |
|  | $h_{cf}\left( t \right)$ | Unlinked cases isolated at time $t$ |
|  | $h_{ct}\left( t \right)$ | Linked cases isolated at time $t$ |

**Table S2** Notified and modelled missed imported wild-type SARS-CoV-2 infections in 2020

| Observed data (●) and modelled outputs (◆) | Time period | | | | | |
| --- | --- | --- | --- | --- | --- | --- |
|  | Jan 18 – Feb 29, 2020 | Mar 1 – Apr 6,  2020 | Apr 7 – Jun 18, 2020 | Jun 19 – Jul 12, 2020 | Jul 13 – Dec 31, 2020 | Apr 1 – May 12, 2021 |
| SARS-CoV-2 lineage | Wild-type | | | | | Delta variant |
| ● Notified imported cases | 29 | 547 | 5 | 82 | 1,537 | 843 |
| ◆ Missed imported infections | 30  (10–60) | 260  (88–2,900) | 0 | 50  (10–200) | 200  (60–1,000) | 200  (50–1,200) |
| ◆ Missed infections per notified imported case | 0.9  (0.4–2) | 0.5  (0.1–2) | 0 | 0.6  (0.1–3) | 0.2  (0.04–0.8) | 0.3  (0.05–1) |

**Table S3** Summary of observed data and modelled outputs (median and 95%CI in parenthesis) by respective time periods in 2020 for wild-type SARS-CoV-2 transmission without using case linkage information for model fitting

| Observed data (●) and modelled outputs (◆) | Time period in 2020 | | | | | | | | | | |  |
| --- | --- | --- | --- | --- | --- | --- | --- | --- | --- | --- | --- | --- |
|  | Overall Jan – Dec | Jan 18 – Feb 29 | Mar 1 – Apr 6 | | Apr 7 – Jun 18 | | Jun 19 – Jul 12 | | Jul 13 – Dec 31 | | |  |
| ● Imported cases | | | | | | | | | |  |  |  |
| Isolated for testing on  Arrival or quarantined | 1,653 | 0 | 50 | 5 | | 78 | | 1,520 | | |  |  |
| Not quarantined | 547 | 29 | 497 | 0 | | 4 | | 17 | | |  |  |
| ● Local cases (by time of isolation | | | | | | | | | |  |  |  |
| Linked | 1,505 | 65 | 606 | | 610 | | 113 | | 111 | | |  |
| Unlinked | 864 | 20 | 204 | | 420 | | 107 | | 113 | | |  |
| ◆ Missed cases | 15,000  (8,400–38,000) | 80  (20–300) | 1,900  (600–10,000) | | 9,300  (5,600–22,500) | | 1,300  (600–3,100) | | 1,700  (900–2,900) | | |  |
| ◆ Total cases (adjusted by time of infection and missed cases) | 17,000  (11,000–41,000) | 200  (100–500) | 3,200  (1,600–12,000) | | 10,000  (6,300–24,000) | | 1,600  (800–3,400) | | 1,900  (1,100–3,200) | | |  |
| ● ICU cases (by time of isolation) | 86 | 13 | 44 | | 28 | | 1 | | 0 | | | |
| ● Deaths (by time of isolation) | 22 | 2 | 11 | | 9 | | 0 | | 0 | | | |
| ◆ Case ICU risk (%) | 3.1  (2.2–4.0) | 22.6  (14.9–31.5) | 3.6  (2.6–4.8) | | 1.7  (1.1–2.5) | | 0.4  (0.3–0.5) | | 0  (0–0) | | | |
| ◆ Infection ICU risk (%) | 0.5  (0.2–0.8) | 12.5  (5.1–21.5) | 1.4  (0.4–2.9) | | 0.2  (0.07–0.2) | | 0.06  (0.03–0.1) | | 0  (0–0) | | | |
| ◆ Case fatality ratio (%) | 0.8  (0.6–1.0) | 3.7  (2.4–5.1) | 1.1  (0.8–1.5) | | 0.4  (0.3–0.6) | | 0  (0–0) | | 0  (0–0) | | | |
| ◆ Infection fatality ratio (%) | 0.1  (0.05–0.2) | 2.0  (0.8–3.5) | 0.4  (0.1–0.9) | | 0.04  (0.02–0.06) | | 0  (0–0) | | 0  (0–0) | | | |


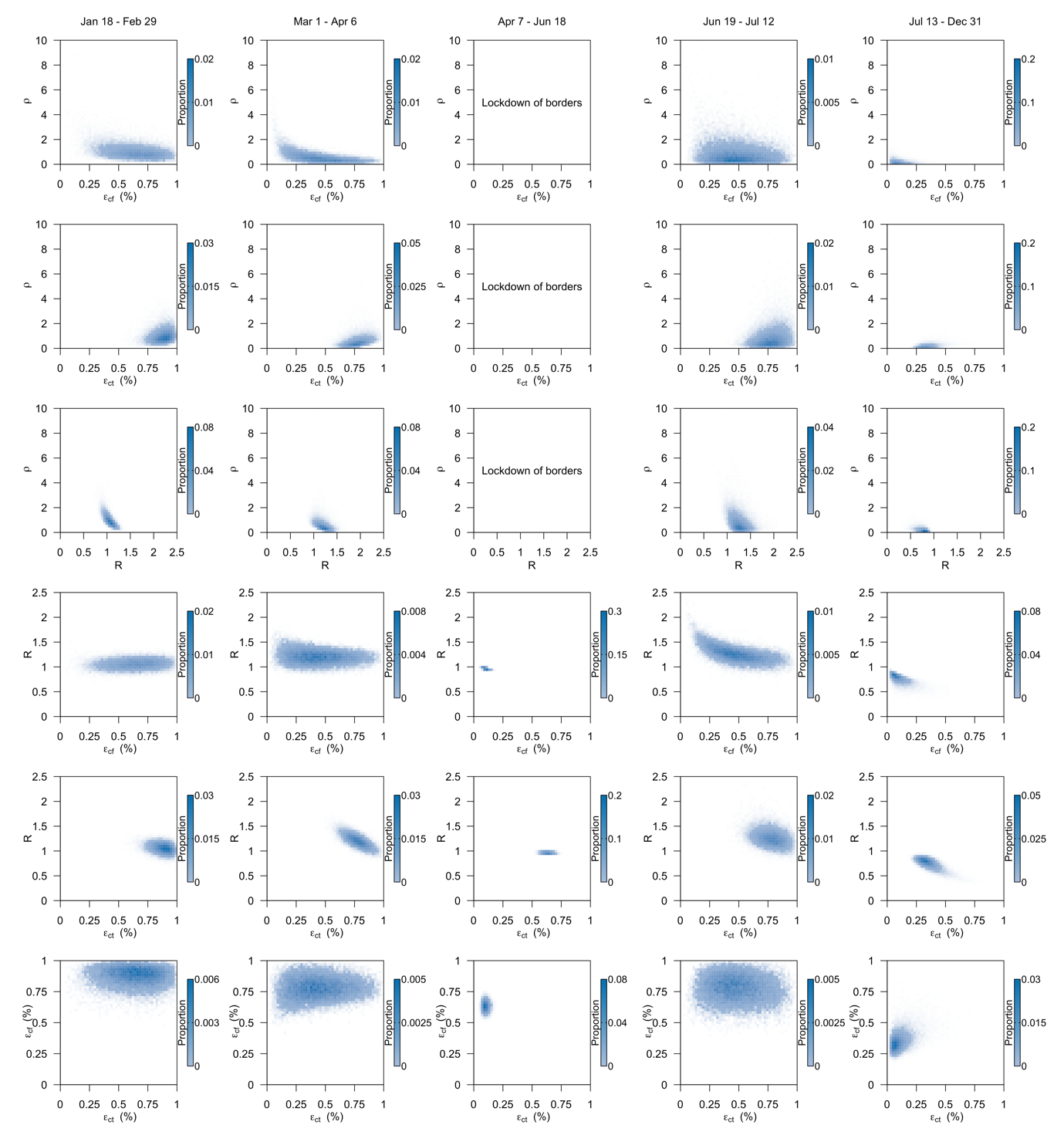


**Figure S1** Contour plots to show the correlation between model parameters. Model parameters were discretised along a range of values and the proportion of posterior samples that falls within a set of values in each pair of parameters was evaluated to derive the plots.


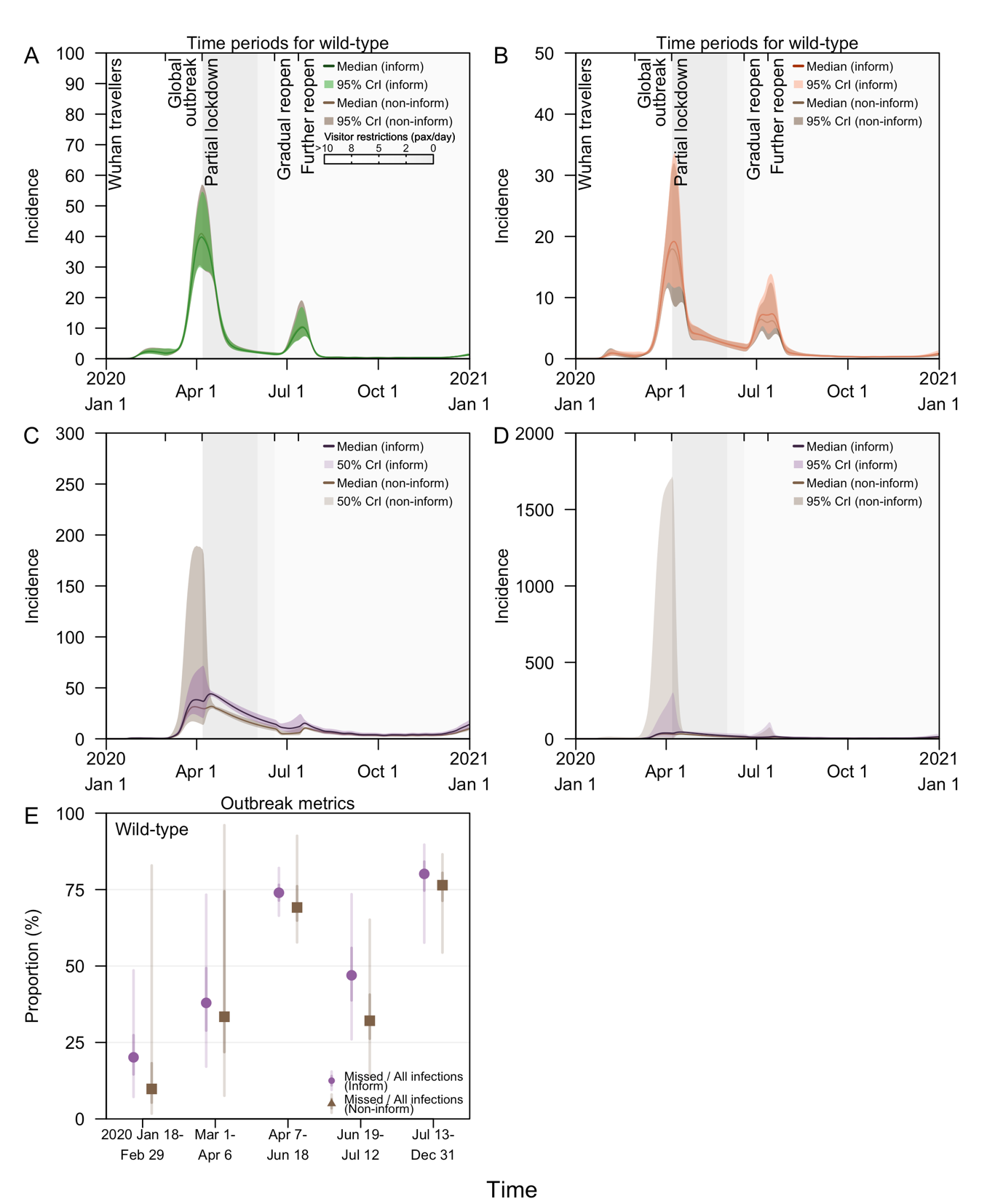


**Figure S2** Posterior estimates for model fitted to time series of linked and unlinked SARS-CoV-2 wild type cases in 2020 using informative and non-informative priors. (A) Incidence of linked cases, (B) incidence of unlinked cases, (C) incidence of missed cases with 50% CI, (D) incidence of missed cases with 95%CI, (E) proportion of missed infections to all infections.

**
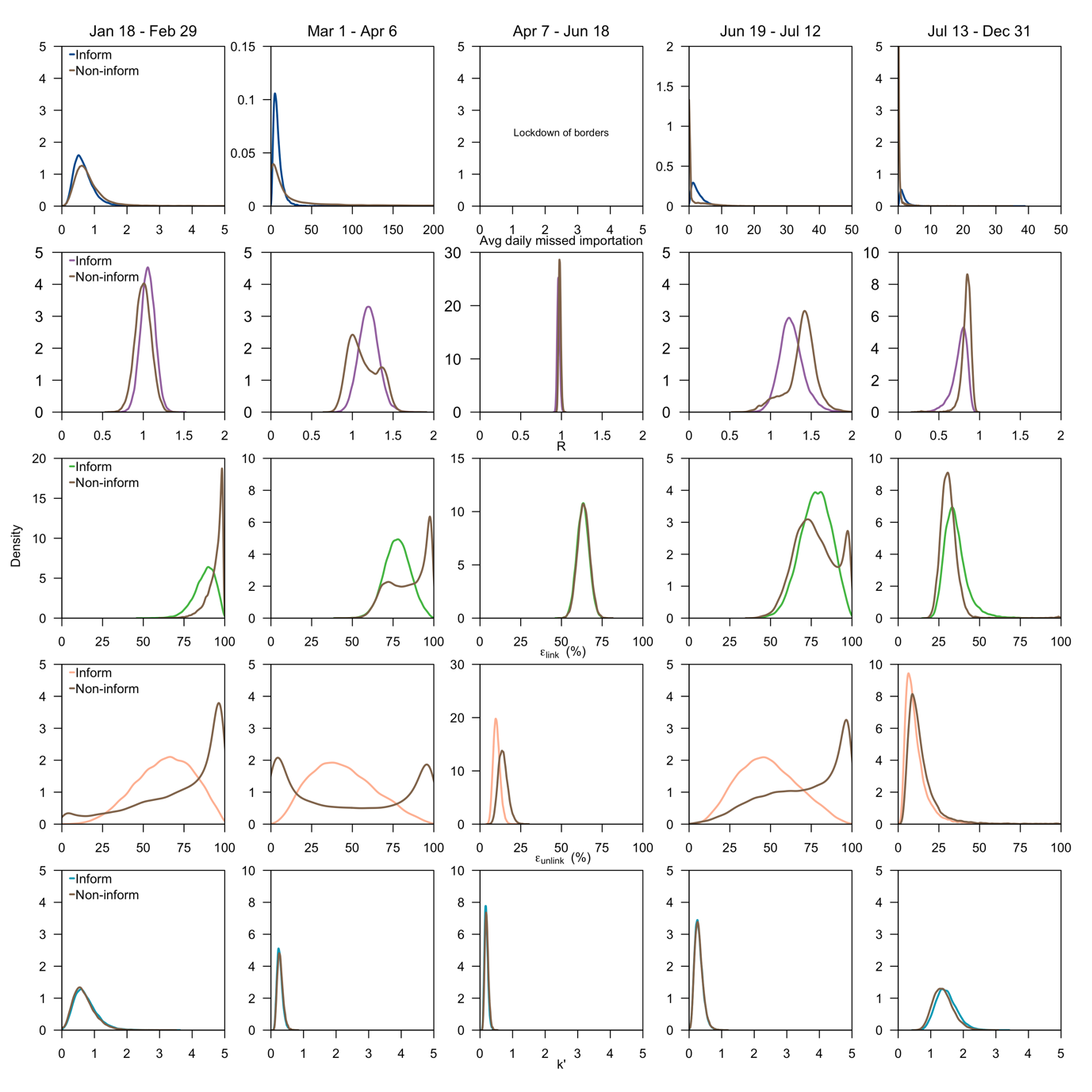
 Figure S3** Posterior density of the parameters for model fitted to time series of linked and unlinked SARS-CoV-2 wild type cases in 2020 using informative (blue: average missed imported cases per day, purple: reproduction number, green: effectiveness of contact tracing, orange: effectiveness of contact tracing, turquoise: dispersion parameter) and non-informative priors (brown).


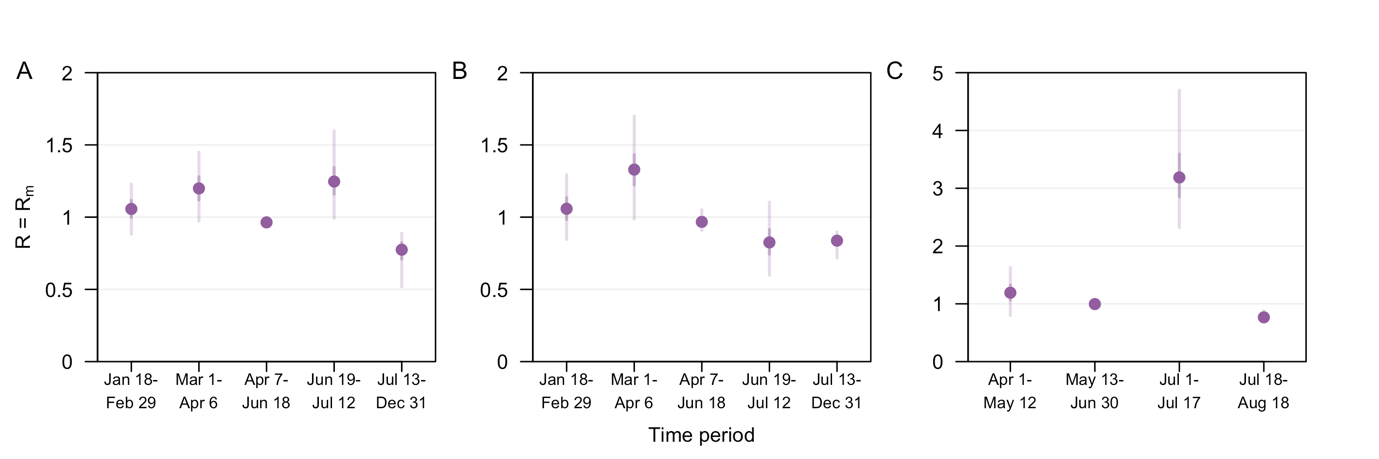


**Figure S4** Reproduction number, R of a SARS-CoV-2 (A,B) wild-type in 2020 and (C) Delta variant case in 2021. (A) using linked and unlinked notified cases for modelling fitting and (B,C) using notified cases with no information of the case linkage for model fitting. Posterior median (dot), 50% CI (dark vertical lines and 95% CI (light vertical lines) as shown.


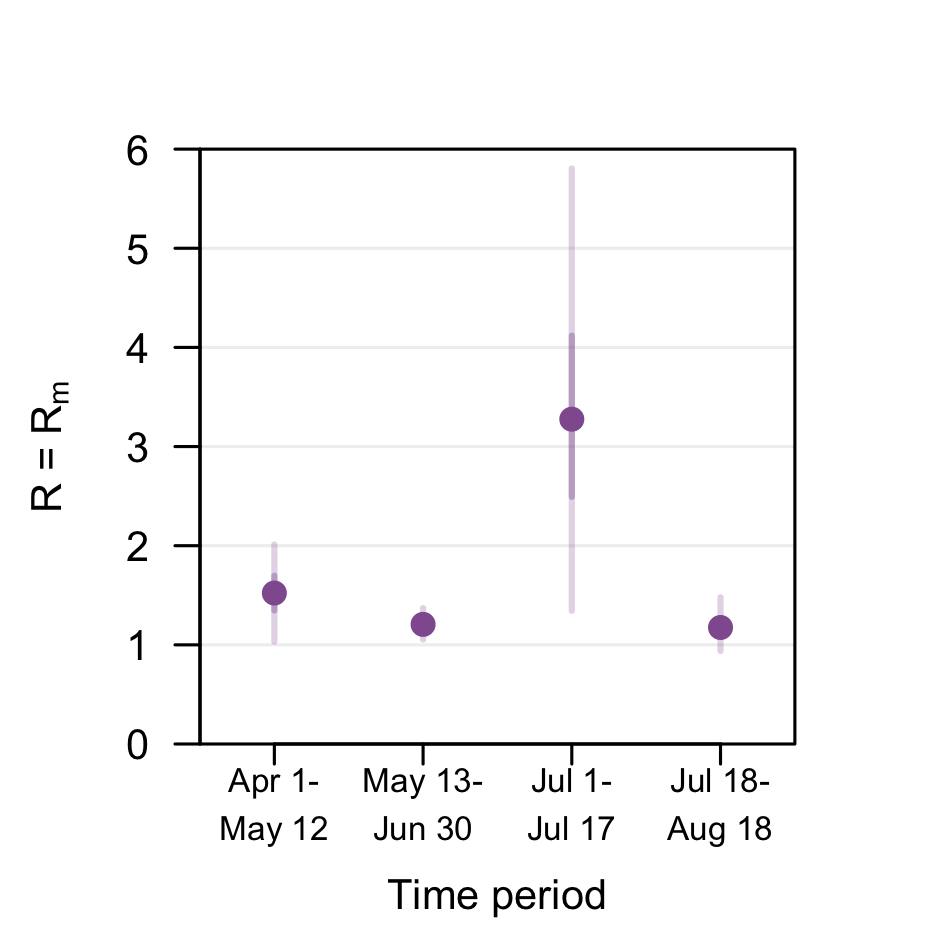


**Figure S5** Reproduction number, R of a SARS-CoV-2 Delta variant case in 2021 after adjusting for vaccine coverage and vaccine effectiveness and using notified cases with no information of the case linkage for model fitting. Posterior median (dot), 50% CI (dark vertical lines and 95% CI (light vertical lines) as shown.


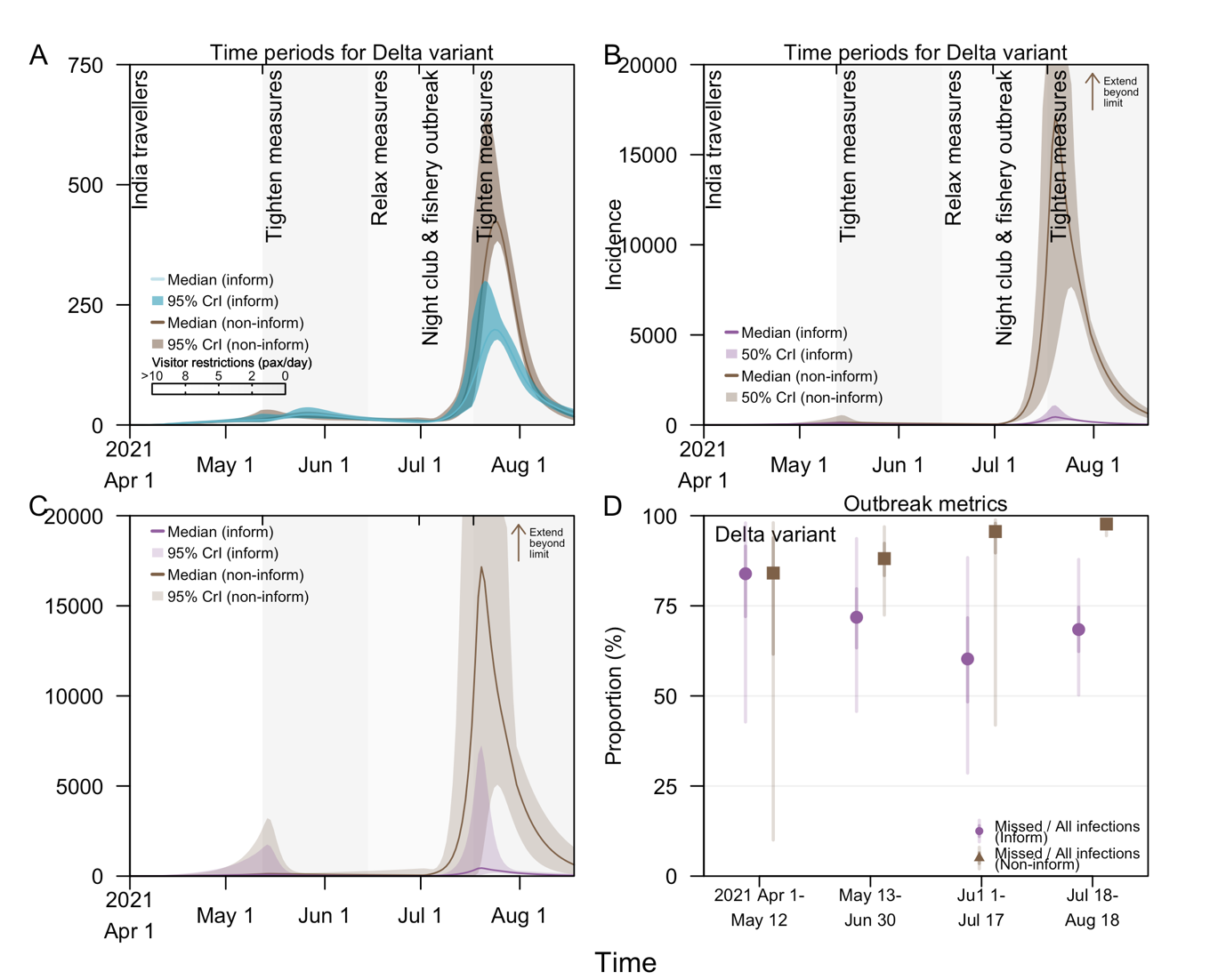


**Figure S6** Posterior estimates for model fitted to time series of SARS-CoV-2 Delta variant cases (without accounting for case linkage) in 2021 using informative and non-informative priors. (A) Incidence of cases, (B) incidence of missed cases with 50% CI, (C) incidence of missed cases with 95%CI, (D) proportion of missed infections to all infections.


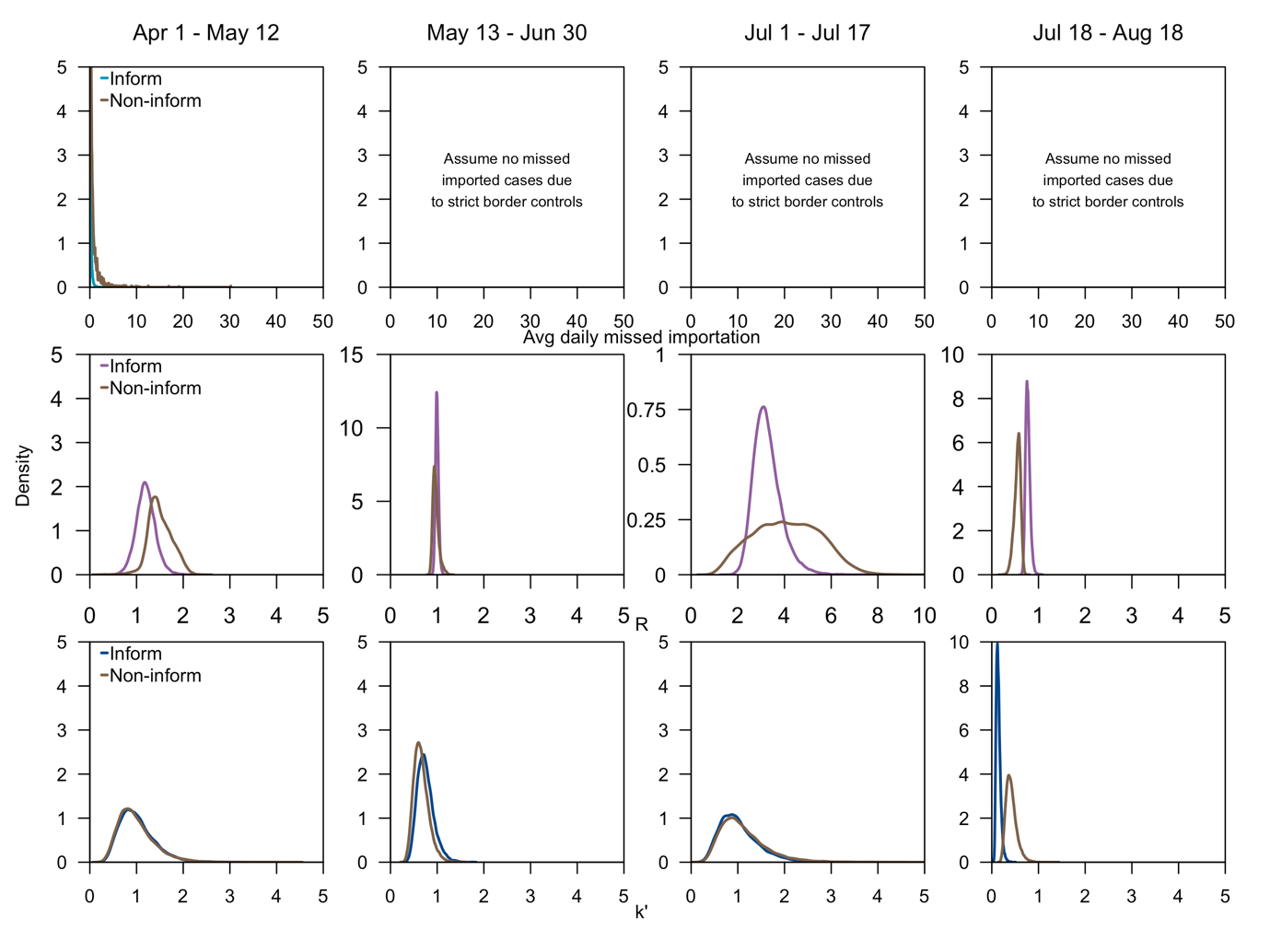


**Figure S7** Posterior density of the parameters for model fitted to time series of linked and unlinked SARS-CoV-2 Delta variant cases (without accounting for case linkage) in 2021 using informative (turquoise: average missed imported cases per day, purple: reproduction number, blue: dispersion parameter) and non-informative priors (brown).


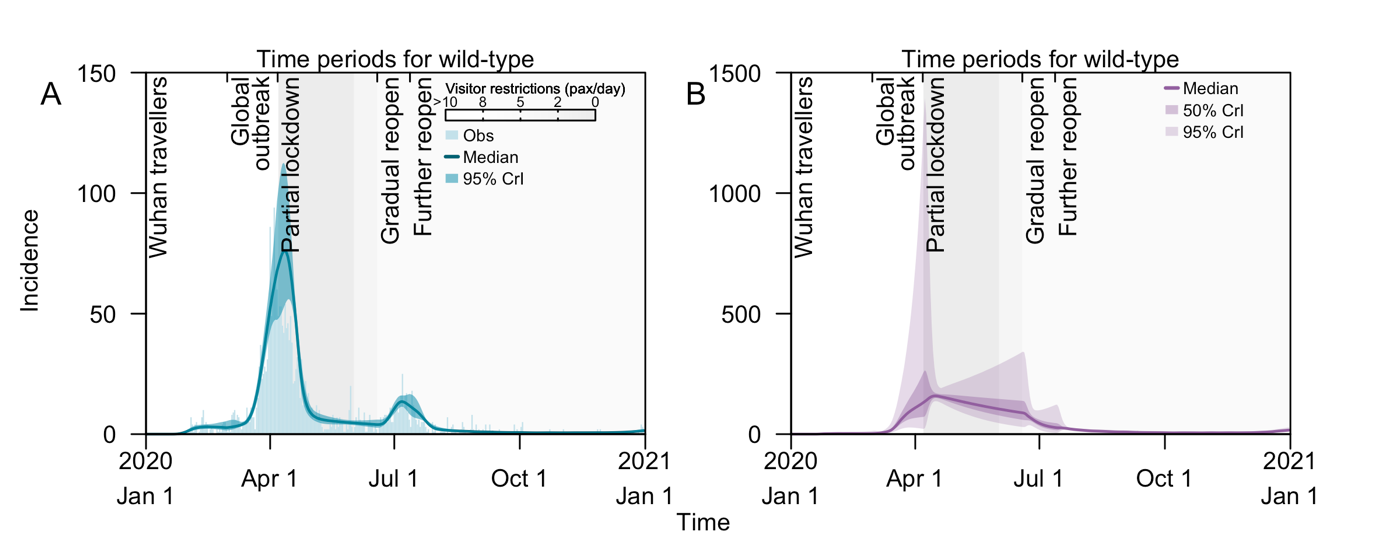


**Figure S8** Daily incidence of COVID-19 cases in Singapore arising from SARS-CoV-2 wild-type transmission in 2020, (A) notified local cases and modelled posteriors, (B) modelled posteriors for local missed infections. Grey shaded areas represents periods with movement and visitor restrictions with darker shades signifying reduced number of visitors to each household per day.

**
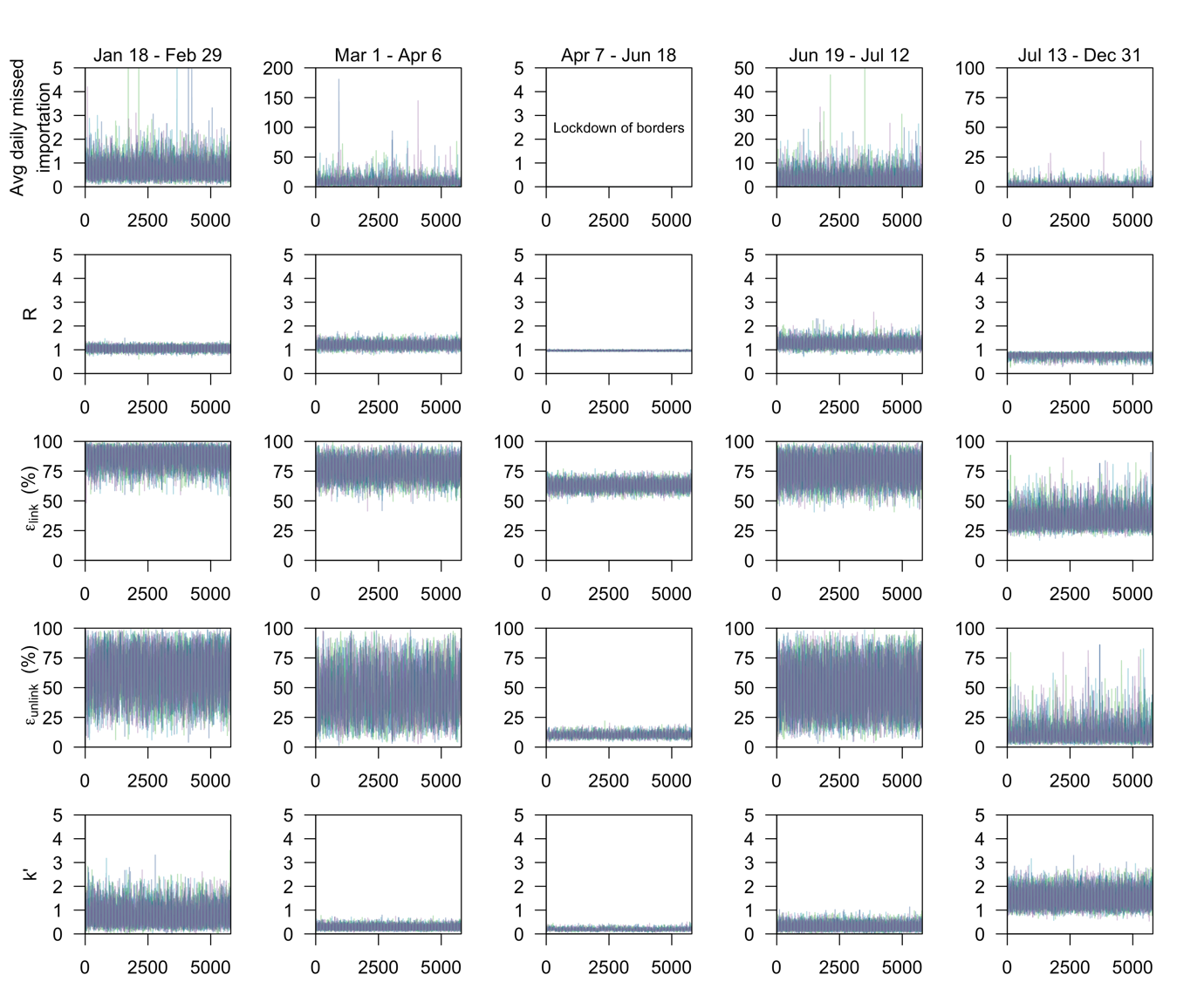
 Figure S9** Markov chain Monte Carlo trace plots for parameters modelling wild-type SARS-CoV-2 transmission in 2020. Different lines represent different MCMC chains.


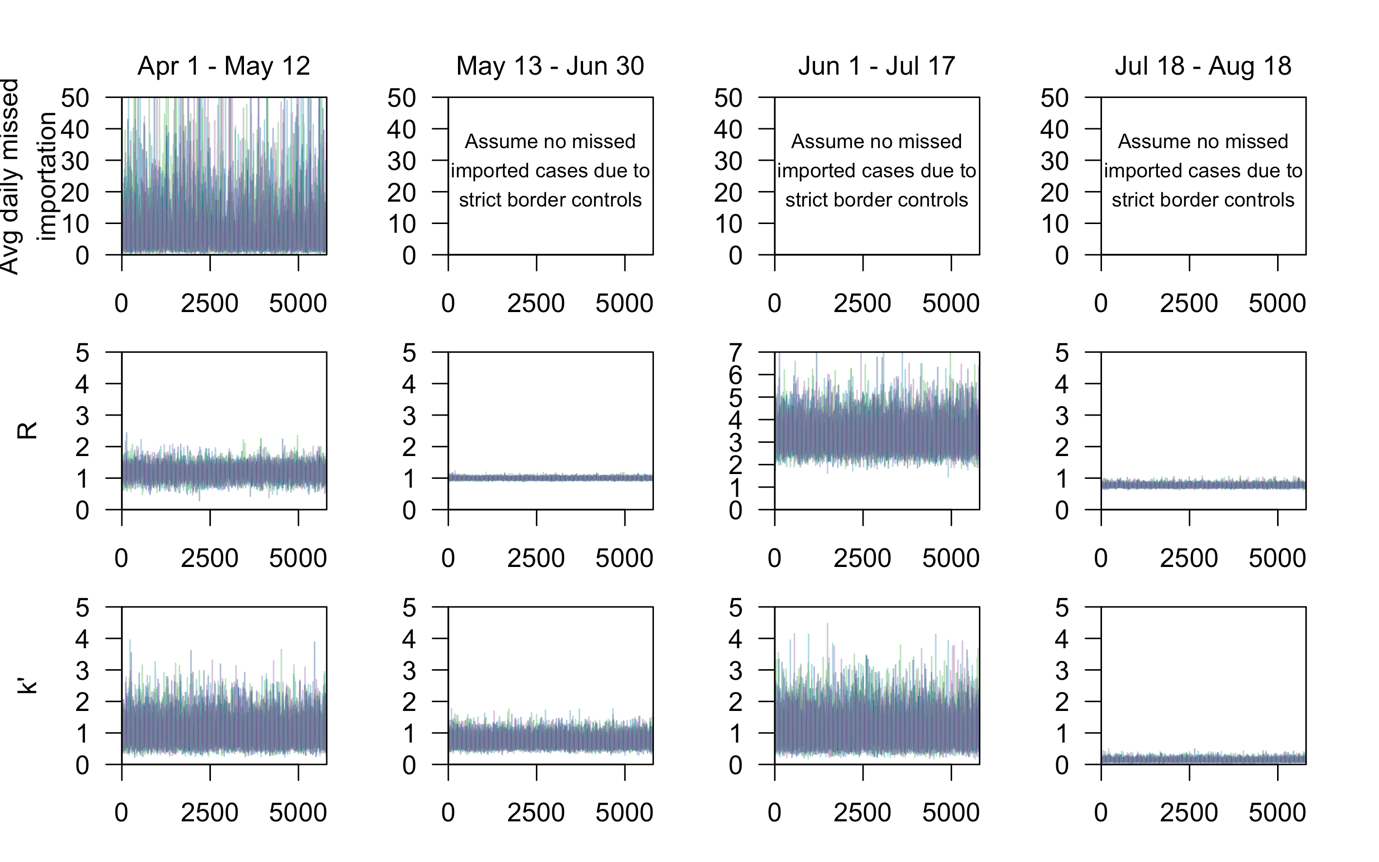
 **Figure S10** Markov chain Monte Carlo trace plots for parameters modelling SARS-CoV-2 Delta variant transmission in 2021. Different lines represent different MCMC chains.
